# Association of Plasma Phospholipid n-3 and n-6 Polyunsaturated Fatty Acids with Type 2 Diabetes: The EPIC-InterAct Case-Cohort Study

**DOI:** 10.1371/journal.pmed.1002094

**Published:** 2016-07-19

**Authors:** Nita G. Forouhi, Fumiaki Imamura, Stephen J. Sharp, Albert Koulman, Matthias B. Schulze, Jusheng Zheng, Zheng Ye, Ivonne Sluijs, Marcela Guevara, José María Huerta, Janine Kröger, Laura Yun Wang, Keith Summerhill, Julian L. Griffin, Edith J. M. Feskens, Aurélie Affret, Pilar Amiano, Heiner Boeing, Courtney Dow, Guy Fagherazzi, Paul W. Franks, Carlos Gonzalez, Rudolf Kaaks, Timothy J. Key, Kay Tee Khaw, Tilman Kühn, Lotte Maxild Mortensen, Peter M. Nilsson, Kim Overvad, Valeria Pala, Domenico Palli, Salvatore Panico, J. Ramón Quirós, Miguel Rodriguez-Barranco, Olov Rolandsson, Carlotta Sacerdote, Augustin Scalbert, Nadia Slimani, Annemieke M. W. Spijkerman, Anne Tjonneland, Maria-Jose Tormo, Rosario Tumino, Daphne L. van der A, Yvonne T. van der Schouw, Claudia Langenberg, Elio Riboli, Nicholas J. Wareham

**Affiliations:** 1 MRC Epidemiology Unit, University of Cambridge, Cambridge, United Kingdom; 2 MRC Human Nutrition Research, Cambridge, UK; 3 Department of Molecular Epidemiology, German Institute of Human Nutrition Potsdam-Rehbruecke, Germany; 4 University Medical Center Utrecht, Utrecht, the Netherlands; 5 Navarre Public Health Institute (ISPN), Pamplona, Spain; 6 CIBER Epidemiología y Salud Pública (CIBERESP), Spain; 7 Department of Epidemiology, Murcia Regional Health Council, IMIB-Arrixaca, Murcia, Spain; 8 Wageningen University, The Netherlands; 9 Inserm, CESP, U1018, Villejuif, France; 10 Univ Paris-Sud, UMRS 1018, Villejuif, France; 11 Gustave Roussy Institute, Villejuif, France; 12 Public Health Division of Gipuzkoa, San Sebastian, Spain; 13 Instituto BIO-Donostia, Basque Government, San Sebastian, Spain; 14 German Institute of Human Nutrition Potsdam-Rehbruecke, Germany; 15 Lund University, Malmö, Sweden; 16 Umeå University, Umeå, Sweden; 17 Catalan Institute of Oncology (ICO), Barcelona, Spain; 18 German Cancer Research Centre (DKFZ), Heidelberg, Germany; 19 University of Oxford, Oxford, United Kingdom; 20 University of Cambridge, Cambridge, United Kingdom; 21 Department of Cardiology, Aalborg University Hospital, Aalborg, Denmark; 22 Department of Public Health, Section for Epidemiology, Aarhus University, Aarhus, Denmark; 23 Aalborg University Hospital, Aalborg, Denmark; 24 Epidemiology and Prevention Unit, Fondazione IRCCS Istituto Nazionale dei Tumori, Milan, Italy; 25 Cancer Research and Prevention Institute (ISPO), Florence, Italy; 26 Dipartimento di Medicina Clinica e Chirurgia, Federico II University, Naples, Italy; 27 Public Health Directorate, Asturias, Spain; 28 Escuela Andaluza de Salud Pública. Instituto de Investigación Biosanitaria ibs.GRANADA. Hospitales Universitarios de Granada/Universidad de Granada, Granada, Spain; 29 Unit of Cancer Epidemiology, Citta' della Salute e della Scienza Hospital-University of Turin and Center for Cancer Prevention (CPO), Turin, Italy; 30 Human Genetics Foundation (HuGeF), Turin, Italy; 31 International Agency for Research on Cancer, Lyon, France; 32 National Institute for Public Health and the Environment (RIVM), Bilthoven, The Netherlands; 33 Danish Cancer Society Research Center, Copenhagen, Denmark; 34 Department of Health and Social Sciences, Universidad de Murcia, Murcia, Spain; 35 Cancer Registry and Histopathology Unit, Civic and M.P.Arezzo Hospital, ASP Ragusa, Italy; 36 School of Public Health, Imperial College London, London, United Kingdom; Chinese University of Hong Kong, CHINA

## Abstract

**Background:**

Whether and how n-3 and n-6 polyunsaturated fatty acids (PUFAs) are related to type 2 diabetes (T2D) is debated. Objectively measured plasma PUFAs can help to clarify these associations.

**Methods and Findings:**

Plasma phospholipid PUFAs were measured by gas chromatography among 12,132 incident T2D cases and 15,919 subcohort participants in the European Prospective Investigation into Cancer and Nutrition (EPIC)-InterAct study across eight European countries. Country-specific hazard ratios (HRs) were estimated using Prentice-weighted Cox regression and pooled by random-effects meta-analysis. We also systematically reviewed published prospective studies on circulating PUFAs and T2D risk and pooled the quantitative evidence for comparison with results from EPIC-InterAct. In EPIC-InterAct, among long-chain n-3 PUFAs, α-linolenic acid (ALA) was inversely associated with T2D (HR per standard deviation [SD] 0.93; 95% CI 0.88–0.98), but eicosapentaenoic acid (EPA) and docosahexaenoic acid (DHA) were not significantly associated. Among n-6 PUFAs, linoleic acid (LA) (0.80; 95% CI 0.77–0.83) and eicosadienoic acid (EDA) (0.89; 95% CI 0.85–0.94) were inversely related, and arachidonic acid (AA) was not significantly associated, while significant positive associations were observed with γ-linolenic acid (GLA), dihomo-GLA, docosatetraenoic acid (DTA), and docosapentaenoic acid (n6-DPA), with HRs between 1.13 to 1.46 per SD. These findings from EPIC-InterAct were broadly similar to comparative findings from summary estimates from up to nine studies including between 71 to 2,499 T2D cases. Limitations included potential residual confounding and the inability to distinguish between dietary and metabolic influences on plasma phospholipid PUFAs.

**Conclusions:**

These large-scale findings suggest an important inverse association of circulating plant-origin n-3 PUFA (ALA) but no convincing association of marine-derived n3 PUFAs (EPA and DHA) with T2D. Moreover, they highlight that the most abundant n6-PUFA (LA) is inversely associated with T2D. The detection of associations with previously less well-investigated PUFAs points to the importance of considering individual fatty acids rather than focusing on fatty acid class.

## Abstract 2

Using a large European cohort, Nita Forouhi and colleagues investigate the association between the concentration of polyunsaturated fatty acids measured in plasma and risk of developing type 2 diabetes.

## Author Summary

**Why Was This Study Done?:**

- Most dietary guidelines recommend the consumption of polyunsaturated fatty acids for cardiovascular health, but it is unclear whether or how n-3 and n-6 types of polyunsaturated fatty acids are related to type 2 diabetes.
- Health concerns have been raised previously about a diet high in linoleic acid (n-6 fatty acid), but its association with type 2 diabetes is unclear.
- Major limitations in previous studies have included the error-prone subjective assessment of the habitual consumption of polyunsaturated fatty acids when dietary intakes were evaluated and a small number of type 2 diabetes cases (*n* = 71 to 673) when objective biomarkers of polyunsaturated fatty acids were measured.

**What Did the Researchers Do and Find?:**

- We measured circulating individual polyunsaturated fatty acids in the blood samples of individuals within a large study from across eight countries of Europe among a reference sample of 15,919 individuals as well as 12,132 individuals who subsequently developed diabetes. Individuals were followed up for an average of approximately 10 y.
- We investigated the association between individual polyunsaturated fatty acids and the risk of future type 2 diabetes using statistical analyses that accounted for factors that could be potential alternative explanations for any observed associations.
- We found that higher levels of blood alpha-linolenic acid, a plant-origin n-3 fatty acid, and n-6 linoleic acid, the most abundant type of polyunsaturated fatty acid, were associated with a lower risk of future type 2 diabetes. In contrast, higher levels of four other minor individual n-6 fatty acids were associated with higher type 2 diabetes risk, while the blood marine-origin n-3 fatty acids were not associated with future diabetes.

**What Do These Findings Mean?:**

- Our findings show that it is important to consider individual circulating polyunsaturated fatty acids for association with type 2 diabetes risk, rather than placing emphasis on the class of circulating polyunsaturated fatty acids.
- We found that blood n-6 linoleic acid, the most abundant polyunsaturated fatty acid, is inversely associated with type 2 diabetes.
- We found no evidence that blood total n-6 polyunsaturated fatty acids may elevate the risk of type 2 diabetes, but several individual minor blood n-6 polyunsaturated fatty acids were associated with increased type 2 diabetes risk, highlighting the importance of polyunsaturated fatty acid metabolism in the development of type 2 diabetes.

## Introduction

Recognition of the importance of dietary fat quality rather than a focus on total fat intake has led to the promotion of dietary guidelines that encourage the consumption of polyunsaturated fatty acids (PUFAs) for cardiometabolic health [1]. Dietary n-3 (omega-3) PUFAs, including alpha-linolenic acid (ALA, 18:3n-3) from plant sources and eicosapentaenoic acid (EPA, 20:5n-3), docosahexaenoic acid (DHA, 22:6n-3), and docosapentaenoic acid (DPA, 22:5n-3) from fish or seafood sources, are postulated to be beneficial for the prevention of type 2 diabetes (T2D), but there is inconclusive evidence from both intervention and observational studies. For instance, n-3 PUFA supplementation with EPA, DHA, or fish oil did not significantly improve glycaemic or insulin markers in randomised trials among general populations or among those with T2D [2,3]. Four separate meta-analyses of prospective studies of the association between dietary long-chain n-3 PUFAs or fish intake, a major source of n-3 PUFAs, and incidence of T2D reported heterogeneous findings with no association, higher risk, or lower risk depending on geographical location [4–7]. Dietary n-6 (omega-6) PUFAs, although they are about 5-fold to 7-fold more abundant in the Western diet than n-3 PUFAs [8,9], have not been systematically examined, and the findings are inconsistent for association with T2D [10–13]. There has also been an ongoing debate about whether dietary linoleic acid (LA; 18:2n6), the most abundant n-6 PUFA, has adverse health effects and therefore whether its consumption should be limited in the population [14–16].

To our knowledge, there is no trial assessing effects of long-term exposure to n-3 or n-6 PUFAs on incidence of T2D, while past evidence from observational studies is limited by accuracy in measurement of habitual PUFA consumption from self-report of dietary intake of foods or supplements. Few studies have examined the associations of objectively measured circulating blood PUFAs with incidence of T2D, with limited sample sizes and the inclusion of a variable number of individual PUFAs [9,17–20].

We aimed to investigate the associations between objectively measured individual n-3 and n-6 PUFAs in the plasma phospholipid fraction and incident T2D in the European Prospective Investigation into Cancer and Nutrition (EPIC)-InterAct case-cohort study [21]. As a secondary objective, to consider the totality of existing evidence, we also conducted a systematic literature review and performed a meta-analysis to compare with the findings from EPIC-InterAct.

## Methods

### Study Design and Population

The methods of the EPIC-InterAct project have previously been described in detail [21]. From among 340,234 persons with 3.99 million person-years of follow-up (1991–2007) in the eight countries of the EPIC study, we ascertained 12,403 T2D cases and selected a random subcohort of 16,835 individuals with baseline plasma samples. After exclusions (*n* = 548 prevalent diabetes; *n* = 133 uncertain diabetes status), the subcohort retained 16,154 individuals and included 778 individuals with incident T2D during follow-up (a feature of the case-cohort design) [21]. From the total 27,779 participants, we excluded 483 without blood fatty acid data, therefore including 12,132 T2D cases and 15,919 subcohort participants in the analysis (with 755 incident cases included by design within the subcohort), with a mean follow-up of 9.8 y. All participants gave written informed consent, and the study was approved by the local ethics committees in the participating countries and the Internal Review Board of the International Agency for Research on Cancer.

### T2D Case Adjudication

Incident T2D was ascertained until 31 December 2007 by reviewing multiple sources of evidence, reported previously [21]: self-report, linkage to primary-care registers and secondary-care registers, medication use, hospital admissions, and mortality data. No diabetes cases were ascertained solely by self-report, and we sought further evidence for cases with information on incident T2D from fewer than two independent sources.

### Measurement of Plasma Phospholipid Fatty Acids

**Fig 1:**
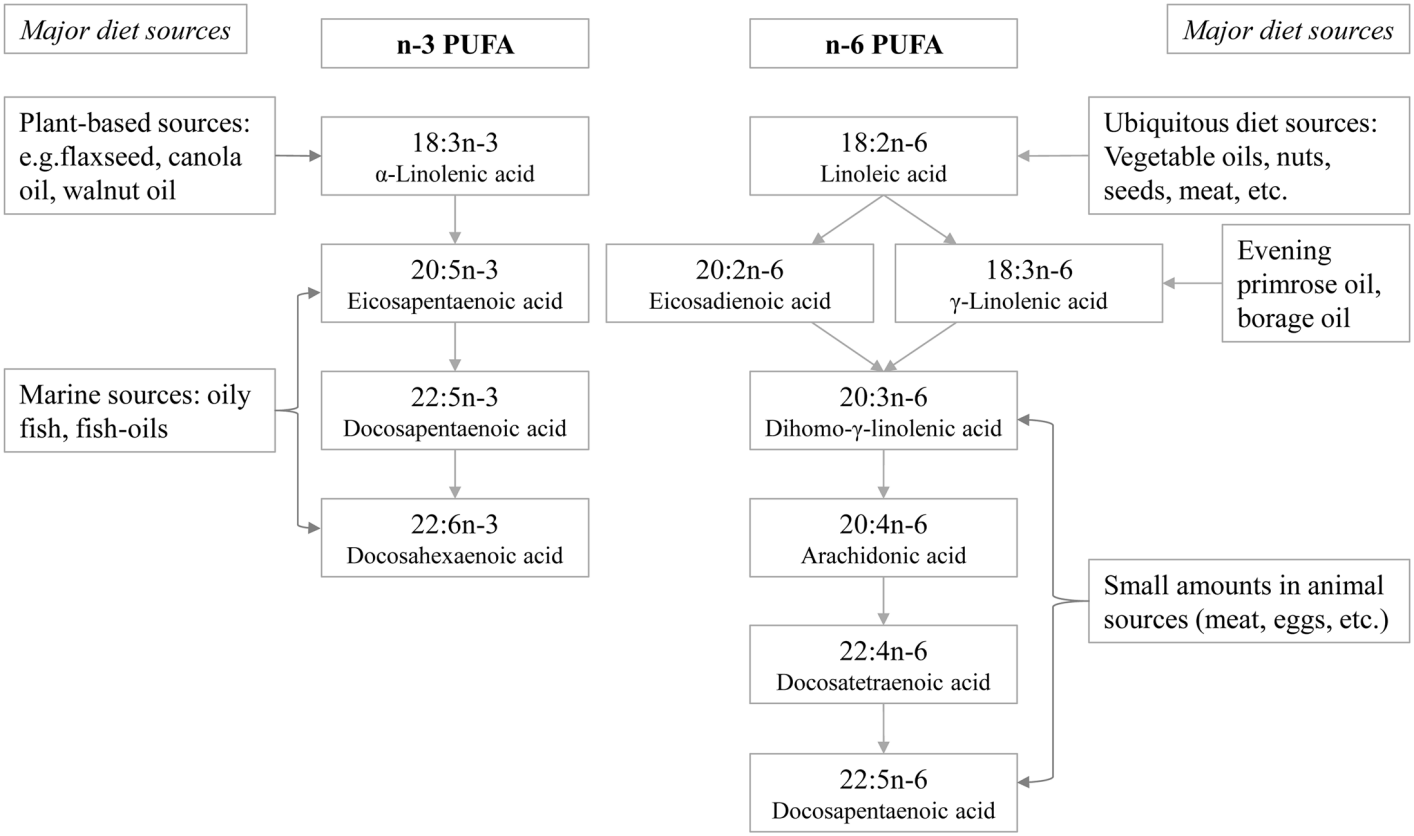
Schematic showing the names of individual n-3 and n-6 PUFAs measured in EPIC-InterAct, their biosynthesis pathways, and major dietary sources.

Fatty acids were profiled at the Medical Research Council Human Nutrition Research, Cambridge, United Kingdom, by analysing plasma samples stored at baseline at −196°C (−150°C in Denmark), a temperature at which fatty acids remain stable [22]. The assay methods were described previously [23]. In short, the plasma phospholipid fraction was obtained using solid phase extraction and hydrolysed and methylated, yielding fatty acid methyl esters (FAME), which were separated by gas chromatography (J&W HP-88, 30 m length) equipped with flame ionisation detection (7890N GC Agilent Technologies, United States). Samples from cases and subcohort participants were processed in a random order by centre, and laboratory staff were blinded to any participant characteristics. Fatty acids were identified by their retention times compared with those of commercial standards and expressed as percent of total phospholipid fatty acids (mol%). Among 27 fatty acids with relative concentrations above 0.05%, we identified 11 individual PUFAs (4 long-chain n-3 PUFAs and 7 n-6 PUFAs) (Fig 1, showing the biosynthesis pathways and major food sources). Human and equine plasma (Sera Laboratories International, UK) were used as quality control samples and included in each batch [23]. The coefficients of variation were less than 8% except for ALA (13%) and γ-linolenic acid (GLA, 21%) [23]. Using the fatty acid measurements, we calculated the sums of total n-3 PUFAs and total n-6 PUFAs. We also estimated the ratios of PUFA variables in the context of biological processes: 18:3n6 / 18:2n6 and 20:4n6 / 20:3n6, indicating estimated activity of Δ6 and Δ5 desaturase enzymes, respectively, catalysing the conversion of LA to GLA and of dihomo-γ-linolenic acid (DGLA) to arachidonic acid (AA); 20:3n6 / 18:2n6, indicating the conversion of LA to DGLA; and ratio of total n-6 PUFAs / total n-3 PUFAs, for its clinical interest in balance between the two major PUFA subclasses.

### Measurement of Covariables at Baseline

Weight and height were measured by trained professionals to standardised protocols, and body mass index (BMI) was calculated as weight divided by squared height (kg/m^2^). Lifestyle questionnaires were used to assess demographics, smoking status, medical history, and education level. Physical activity was assessed by a validated questionnaire from which an ordered four-category variable was derived. Glycated haemoglobin (HbA1c) was measured (SHL, Etten-Leur, Netherlands) in the erythrocyte fraction from samples stored at −196°C (−80°C in Umeå), using the Tosoh-G8 (Tosoh Bioscience, Japan).

### Statistical Analysis

All analyses were performed using Stata, version 13 (Stata, College Station, Texas). We estimated the association with incident T2D of each of the individual n-3 and n-6 PUFAs, total n-3 PUFAs, total n-6 PUFAs, and predefined ratios of PUFA variables. Country-specific hazard ratios (HRs) per standard deviation (SD; calculated in the subcohort) and 95% confidence intervals (95% CI) were estimated using Prentice-weighted Cox regression [21], which allows for over-representation of cases in a case-cohort design, and pooled using random-effects meta-analysis. Heterogeneity between countries was expressed as I^2^ values [24]. We adjusted for potential confounders as follows: Model 1 included age (as underlying timescale), study centre, sex, physical activity index (inactive, moderately inactive, moderately active, or active), smoking status (never, former, or current), education level (none, primary school, technical or professional school, secondary school, or higher education) and BMI (continuous). Model 2 further adjusted for total energy intake (continuous), alcohol (none, >0–<6, 6–<12, 12–<24 and ≥24 g/d), and the other potential dietary confounders unlikely to be major sources of PUFAs: meat, fruits, vegetables, dairy products, and soft drinks (continuous). Model 3 additionally adjusted for possible dietary sources of PUFAs (fish and shellfish, nuts and seeds, vegetable oil, olive oil, and margarine; continuous; Fig 1). In a secondary analysis, we also examined the association of quintiles of each of the PUFA exposures with T2D.

In sensitivity analyses, using Model 3, we examined additional confounding or mediating effects of plasma phospholipid saturated fatty acids (even-chain 14:0, 16:0, and 18:0; and odd-chain 15:0 and 17:0; continuous), prevalent comorbidity (including myocardial infarction, stroke, and cancer; yes/no for each), and baseline HbA1c. To minimise the possibility of reverse causality, we repeated analyses after excluding 2,348 individuals with baseline HbA1c ≥6.5% (≥48 mmol/mol) and after excluding T2D cases (*n* = 1,048) occurring within 2 y after baseline.

### Systematic Review and Meta-analysis of Published Studies

Our objective was to do a comparative meta-analysis of the existing evidence in order to provide a systematic comparison with EPIC-InterAct findings rather than providing a narrative comparison. To compare the findings from EPIC-InterAct with the published literature, we identified prospective studies published by 3 November 2015 that reported on the association of circulating n-3 or n-6 PUFAs with T2D using PubMed (S1 Text for further details). We performed a random effects meta-analysis of the association of circulating n-3 or n-6 PUFAs with T2D from published studies identified as eligible from the literature review, using estimates from a model with the greatest degree of adjustment for potential confounders. The overall effect was estimated per study-specific SD [25] and meta-analysed by lipid fraction and across lipid fractions for each fatty acid. Heterogeneity was assessed using Q and I^2^ statistics [24]. Publication bias was investigated using Egger’s and Begg’s tests.

## Results

**Fig 2:**
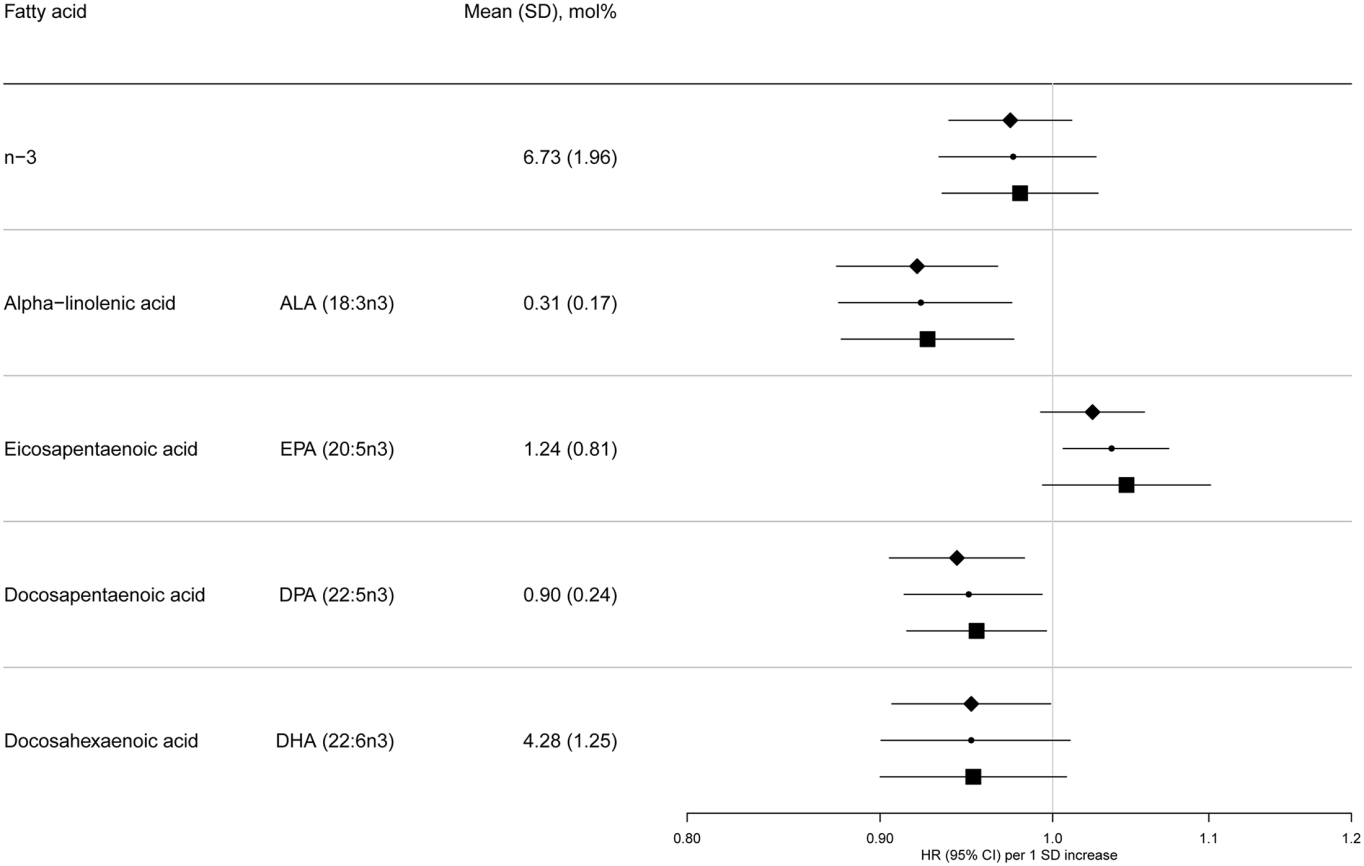
HRs of T2D and 95% CIs per 1 SD increase in total and individual n-3 PUFAs (ALA, EPA, DPA, and DHA): EPIC-InterAct study. Model 1: displayed as diamonds. Age as underlying time variable, and adjusted for centre, sex, physical activity (inactive, moderately inactive, moderately active, or active), smoking (never, former, or current), education level (none, primary school, technical or professional school, secondary school, or higher education), and BMI (continuous, kg/m^2^). Model 2: displayed as circles. Adjusted as in Model 1 + total energy intake (continuous, kcal/d), alcohol (none, >0–<6, 6–<12, 12–<24 and ≥24 g/d), and (continuous, g/d intake of) meat, fruit and vegetables, dairy products, and soft drinks. Model 3: displayed as squares. Adjusted as in Model 2 + (continuous, g/d intake of) fish and shellfish, nuts and seeds, vegetable oil, olive oil, and margarine.

**Fig 3:**
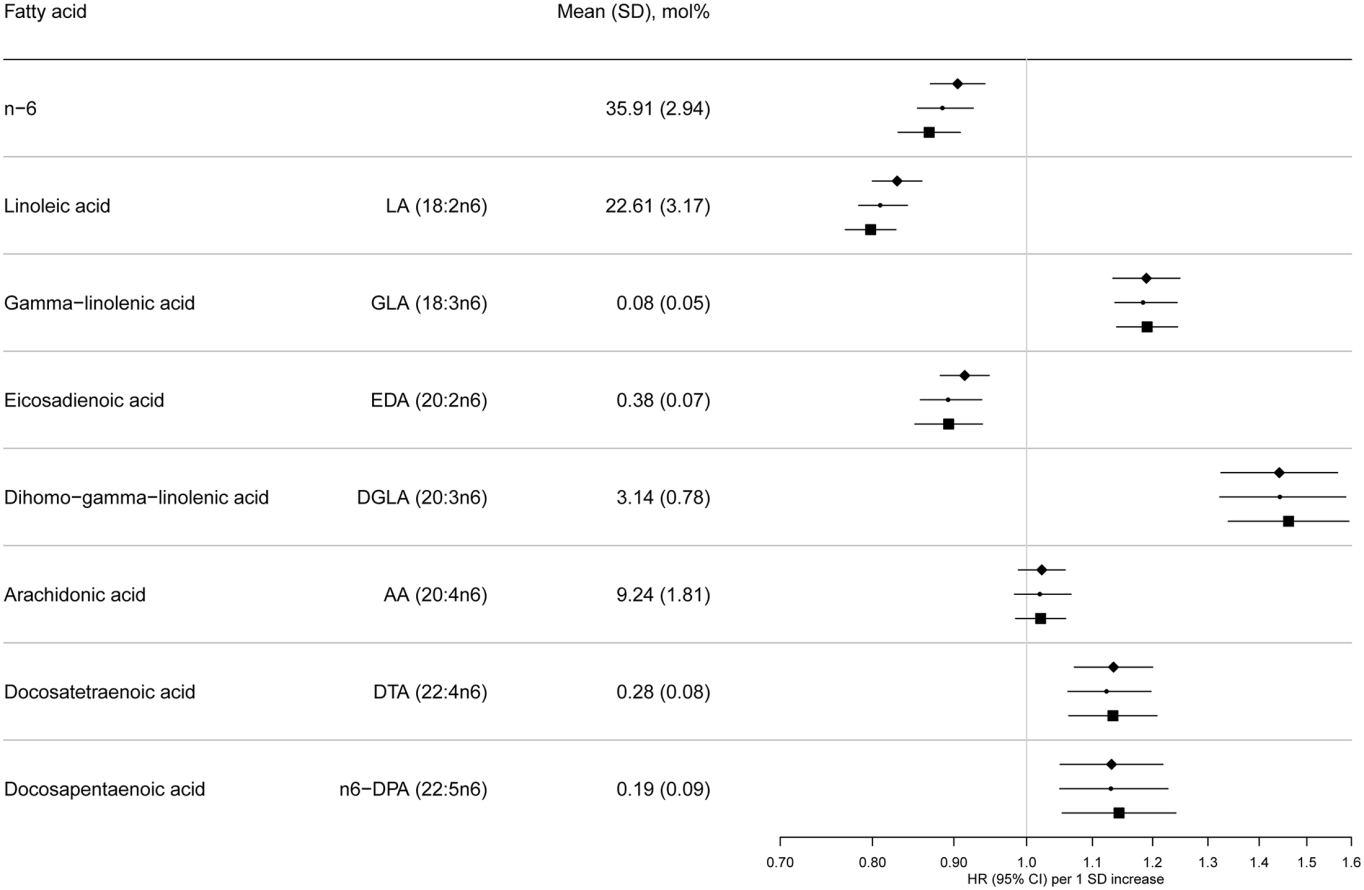
HRs of T2D and 95% CIs per 1 SD increase in total and individual n-6 PUFAs (LA, GLA, EDA, DGLA, AA, DTA, and n-6 DPA): EPIC-InterAct study. Model 1: displayed as diamonds. Age as underlying time variable, and adjusted for centre, sex, physical activity (inactive, moderately inactive, moderately active, or active), smoking (never, former, or current), education level (none, primary school, technical or professional school, secondary school, or higher education), and BMI (continuous, kg/m^2^). Model 2: displayed as circles. Adjusted as in Model 1 + total energy intake (continuous, kcal/d), alcohol (none, >0–<6, 6–<12, 12–<24 and ≥24 g/d), and (continuous, g/d intake of) meat, fruit and vegetables, dairy products, and soft drinks. Model 3: Displayed as squares. Adjusted as in Model 2 + (continuous, g/d intake of) fish and shellfish, nuts and seeds, vegetable oil, olive oil, and margarine.

Table 1 summarises the baseline characteristics by case status, and S1 and S2 Tables show characteristics by age, sex, BMI, and country. Among subcohort participants, PUFAs comprised a mean 42.7±2.1% of the total phospholipid fatty acids, of which n-6 PUFAs were more abundant (35.9±2.9%) than n-3 PUFAs (6.7±2.0%). PUFAs with relatively high concentrations, e.g., >1%, were LA (22%), DGLA (3.1%), AA (9.2%), EPA (1.2%), and DHA (4.3%) (Figs 2 and 3).

**Table 1:** The distribution of sociodemographic and dietary factors at baseline by future case and noncase status: EPIC-InterAct study.

	Noncases		Cases	
	*n* = 15,164		*n* = 12,132	
	**Mean / %**	***n* / SD**	**Mean / %**	***n* / SD**
Age (y)	52.2	9.2	55.6	7.7
BMI (kg/m^2^)	25.8	4.1	29.7	4.7
Sex				
Men	37.1	5,629	49.7	6,030
Women	62.9	9,535	50.3	6,102
Education Level				
None	7.3	3,525	9.7	3,615
Primary	32.0	5,016	41.1	3,918
Technical or professional	22.7	3,388	22.9	2,434
Secondary	15.4	3,028	10.9	1,992
Higher education	20.7		13.0	
Physical Activity		1,101		1,171
Inactive	23.2	4,851	29.8	4,989
Moderately inactive	33.1	3,446	32.3	2,783
Moderately active	22.3	2,336	20.1	1,323
Active	20.0	3,141	16.4	1,574
Smoking Status				
Never	46.7	7,077	40.3	4,895
Former	26.7	4,050	30.8	3,739
Current	25.5	3,860	27.6	3,344
Alcohol Consumption				
None	16.0	2,432	18.3	2,223
>0–<6 g/d	33.7	5,117	32.3	3,924
6–<12 g/d	15.7	2,378	14.0	1,701
12–<24 g/d	16.1	2,439	14.7	1,788
≥24 g/d	18.1	2,751	20.0	2,426
Total Energy Intake (kcal/d)	2,136	631	2,175	671
	**Median**	**IQR**	**Median**	**IQR**
Intake (g/d) of:				
Meat	73.8	(46.1,107.9)	83.8	(53.4,120.9)
Fruit and vegetables	369.5	(236.4,544.6)	355.0	(223.0,537.3)
Soft drinks	3.3	(0,64.7)	9.8	(0,92.4)
Dairy	287.7	(168.0,455.7)	281.1	(159.5,464.7)
Fish and shellfish	28.9	(14.8,51.2)	32.1	(16.1,55.2)
Nuts and seeds	0	(0,1.3)	0	(0,0.4)
Vegetable oil	5.5	(1.2,20.4)	4.9	(0.5,19.8)
Olive oil	0.02	(0,13.9)	0	(0,10.5)
Margarine	5.3	(0.08,21.8)	7.4	(0.09,25.0)

BMI, body mass index; IQR, interquartile range; SD, standard deviation.

The associations we observed did not change substantially by covariables examined (Model 1, 2, and 3) (Fig 2 and S7 Table). There was no evidence of an association between total n-3 PUFAs and T2D, but an inverse association was evident with ALA (18:3n3), with a per 1 SD HR of 0.93 (95% CI 0.88–0.98; Model 3). There was no association with EPA (20:5n3) and DHA (22:6n3), and a borderline inverse association with DPA (22:5n3). Heterogeneity of associations by country appeared to be weak to moderate (I^2^ = 0% to 50%) (S1 Fig).

Sensitivity analyses were not markedly different for the majority of associations (S3 Table). As an exception, n-3 PUFAs were found to be sensitive to additional adjustment, showing a positive association of EPA with T2D [HR 1.09; 95% CI 1.04–1.14] when adjusted for odd-chain saturated fatty acids and an inverse association of DHA (HR 0.92; 95% CI 0.87–0.98] when adjusted for even-chain saturated fatty acids. Secondary analysis of quintiles of n-3 PUFAs showed similar overall patterns (S4 Table).

Associations with n-6 PUFAs are shown in Fig 3. LA (18:2n6) was strongly inversely associated with T2D (per 1 SD HR 0.80; [95% CI 0.77–0.83], Model 3]. AA (20:4n6) was not associated, while four of the individual n-6 PUFAs were positively associated with T2D: Model 3, GLA (18:3n6) (HR 1.19; 95% CI 1.14–1.24], DGLA (20:3n6) (HR 1.46; 95% CI 1.34–1.59), docosatetraenoic acid (DTA, 22:4n6) (HR 1.13; 95% CI 1.06–1.21), and n-6 DPA (22:5n6) (HR 1.14; 95% CI 1.05–1.24). In sensitivity analyses, results were similar, except adjustment for saturated fatty acids altered findings for 22:4n6 and 22:5n6 to the null (S3 Table). Associations by country are shown in S1 Fig. The findings using quintile distributions of n-6 PUFAs were consistent (S4 Table). For instance, comparing the top and bottom quintiles, there was an inverse association with T2D for LA (HR 0.54; 95% CI 0.47–0.61, *p*-trend < 0.001).

The ratio of GLA (18:3n6) to LA (18:2n6), representing estimated Δ6 desaturase activity, was positively associated with T2D (HR 1.21, 95% CI 1.16–1.26; Model 3), while the ratio of AA (20:4n6) to DGLA (20:3n6), representing Δ5 desaturase activity, was inversely associated (HR 0.73; 95% CI 0.67–0.80). The ratio of DGLA to LA was positively associated with T2D (HR 1.44; 95% CI 1.33–1.57), but there was no association of total n6 to n3 ratio with T2D (HR 0.98; 95% CI 0.93–1.04).

The flow diagram for the systematic literature search and study profiles is shown in S2 Fig and S5 Table. The combined findings from the previously published evidence were based on between 71 to a maximum of 2,499 T2D cases from between one to nine studies, depending on individual fatty acids that were measured (Table 2). Compared with the findings in EPIC-InterAct (12,131 T2D cases), the key differences were that each of circulating total n-6 PUFAs, LA, eicosadienoic acid (EDA), DTA and n-6 DPA, as well as the estimated Δ6 desaturase activity, were not significantly associated with T2D. There was no evidence of publication bias for any fatty acid variables (*p*_Begg’s_ and *p*_Egger’s_ > 0.1), except for the 20:3n6/18:2n6 ratio (*p*_Begg_ *=* 0.04 and *p*_Egger_ = 0.003) for which exclusion of a potential outlying estimate retained the significant positive association (S3 Fig). Despite differences in lipid fraction (e.g., plasma or erythrocyte phospholipids or total sera), laboratory setting, and population characteristics, there was little heterogeneity for ALA and GLA (I^2^ = 0) and moderate heterogeneity for major individual PUFAs (EPA, DPA, DHA, and LA) (I^2^ = 30% to 53%), but substantial heterogeneity for total n-3, total n-6, AA, and the other minor PUFAs. The observed heterogeneity was not explained to a significant degree by lipid fraction in metaregression analysis, except for total n-3 and AA where I^2^ as the measure of heterogeneity became 0.0% after adjustment for lipid fractions (S6 Table).

**Table 2:** Results for associations of circulating n-3 and n-6 PUFAs and fatty acid ratios with incidence of T2D: EPIC-InterAct study and comparative meta-analysis of the published prospective studies*.

	HR (95% CI) EPIC-InterAct*	*n* Cases	HR (95% CI) Comparative Meta-analysis*	*n* Cases	*n* Studies	I^2^	*P*-heterogeneity
**n-3 Polyunsaturated Fatty Acids**	0.98 (0.93–1.03)	12,131	0.95 (0.84–1.06)	1,667	5	63.2	0.03
α-Linolenic acid, ALA (18:3n3)	0.93 (0.88–0.98)	12,131	0.96 (0.92–0.99)	2,499	9	0	0.62
Eicosapentaenoic acid, EPA (20:5n3)	1.05 (0.99–1.10)	12,131	0.96 (0.90–1.01)	2,499	9	38.7	0.11
Docosapentaenoic acid, DPA (22:5n3)	0.95 (0.91–1.00)	12,131	0.92 (0.86–0.99)	2,043	7	38.2	0.14
Docosahexaenoic acid, DHA (22:6n3)	0.95 (0.90–1.01)	12,131	0.97 (0.91–1.03)	2,499	9	52.6	0.03
**n-6 Polyunsaturated Fatty Acids**	0.87 (0.83–0.91)	12,131	0.89 (0.77–1.02)	1,667	5	73.9	<0.001
Linoleic acid, LA(18:2n6)	0.80 (0.77–0.83)	12,131	0.86 (0.77–0.95)	1,907	8	50.1	0.05
γ-Linolenic acid, GLA (18:3n6)	1.19 (1.14–1.24)	12,131	1.19 (1.08–1.30)	1,195	4	0	0.43
Eicosadienoic acid, EDA (20:2n6)	0.89 (0.85–0.94)	12,131	0.82 (0.54–1.23)	872	2	86.3	0.01
Dihomo-γ-linolenic acid, DGLA (20:3n6)	1.46 (1.34–1.59)	12,131	1.30 (1.11–1.52)	1,714	6	77.0	<0.001
Arachidonic acid, AA (20:4n6)	1.02 (0.98–1.06)	12,131	1.02 (0.91–1.16)	1,873	7	71.4	<0.001
Docosatetraenoic acid, DTA (22:4n6)	1.13 (1.06–1.21)	12,131	1.15 (0.94–1.41)	903	3	60.2	0.08
Docosapentaenoic acid, n6-DPA (22:5n6)	1.14 (1.05–1.24)	12,131	1.22 (0.97–1.53)	71	1		
**Fatty Acid Ratios**							
Ratio: 18:3n6 / 18:2n6 (Δ6 desaturase)	1.21 (1.16–1.26)	12,131	1.21 (0.97–1.50)	226	2	86.4	<0.001
Ratio: 20:4n6 / 20:3n6 (Δ5 desaturase)	0.73 (0.67–0.80)	12,131	0.75 (0.70–0.80)	948	5	0	0.93
Ratio: 20:3n6/ 18:2n6 (DGLA to LA ratio)	1.46 (1.34–1.59)	12,131	1.52 (1.33–1.73)	722	4	40.9	0.17
Ratio: n6/n3	0.98 (0.93–1.04)	12,131	0.93 (0.80–1.07)	364	1		

* HRs for EPIC-InterAct are per 1 SD of each fatty acid using estimates from Model 3 (see methods). For the comparative literature-based meta-analysis, the HRs are per 1 SD (study specific) of each fatty acid, derived from random-effects meta-analysis based on the model most adjusted for potential confounders in each study. Results for the comparative literature-based meta-analysis were derived from one to nine prospective cohort, case-cohort, or nested case-control studies (S5 Table).

## Discussion

EPIC-InterAct provides the largest and most extensive evaluation to date of the association between 11 individual plasma phospholipid n-3 and n-6 PUFAs and incident T2D. These findings indicate that there is no significant association between total n-3 PUFAs and T2D, but that the plant-derived n-3 PUFA, ALA, is robustly inversely associated with T2D incidence. In contrast, the largely marine-derived n-3 PUFAs were not associated, except for a modest inverse association of DPA with T2D. Total plasma phospholipid n-6 PUFAs were inversely associated, largely driven by the most abundant circulating PUFA, LA. Our findings also highlight that four n-6 PUFAs (GLA, DGLA, DTA, and n-6DPA) were associated with higher T2D incidence, while AA (20:4n6) was not associated with T2D. There was no association of the total n6-to-n3 PUFA ratio, but estimated Δ6 desaturase activity was positively—and Δ5 desaturase activity inversely—associated with T2D.

### Findings in Context: n-3 Fatty Acids

The overall null associations of circulating EPA and DHA with T2D in EPIC-InterAct are consistent with our concurrent comparative meta-analysis of the published literature. Moreover, our findings for circulating EPA and DHA are consistent with the lack of evidence of association between dietary EPA+DHA intakes and T2D risk in prospective cohorts [6] or of net benefit or harm of marine-origin n-3 supplementation on glycaemia or insulin sensitivity in randomised trials [2,3,26]. However, our sensitivity analyses showed that the null findings for EPA and DHA should be interpreted with caution because there was a positive association of T2D with EPA after further adjustment for the odd-chain saturated fatty acids (15:0 and 17:0) and, conversely, an inverse association with DHA after adjustment for even-chain saturated fatty acids (sum of 14:0, 16:0, and 18:0). This indicates that the observed findings may be influenced by complex inter-relationships and metabolic effects of different fatty acids [27], but the totality of current evidence does not support benefits of EPA or DHA for T2D prevention.

On the other hand, we found a modest inverse association between DPA and T2D in EPIC-InterAct as well as in our comparative meta-analysis, though it has been less well investigated previously [6,28]. n-3 DPA is an intermediate between EPA and DHA and appears to have unique biological functions—for example, having a greater potency than EPA and DHA as a precursor of eicosanoids [28]—but little is known of DPA’s glycaemic effects [29,30]. In genome-wide association analyses, gene variants associated with circulating n-3 PUFA were found to explain 11.4% of the variance of DPA, much higher than that for EPA or DHA (<1%) [31], highlighting that DPA levels are related to fatty acid metabolism. Our current findings suggest that there may be a potential beneficial (inverse) association between DPA and T2D, but further biochemical and clinical research is needed to examine a causal role of DPA on glycaemic outcomes and regulation of DPA synthesis.

Our findings provide convincing evidence for an inverse association between the plant food-based circulating ALA and T2D. This is in line with supportive evidence from randomised trials that ALA or flaxseed oil (rich in ALA) intervention could improve glucose homeostasis [32–35] and also in line with prior weak evidence of dietary ALA showing nonsignificant inverse association with T2D with relative risk 0.93 (95% CI 0.83–1.04) [6]. Conversion of ALA (an essential fatty acid from the diet) to long-chain n-3 PUFAs is not likely to explain the observation, because the conversion is small (0.2%–8% of ALA) [36] and because neither EPA nor DHA was associated with T2D. The precise mechanisms of how ALA may influence diabetes risk are unknown, but there is evidence suggesting that ALA may induce insulin secretion through direct actions on G-protein receptors expressed in pancreatic β-cells and through stimulating enteroendocrine L-cells, as well as by enhancing insulin sensitivity through hepatic insulin-like growth factor-1 (IGF-1) related pathways [37].

### Findings in Context: n-6 Fatty Acids

EPIC-InterAct findings provide convincing evidence for a strong inverse association between circulating LA (18:2n6), the most abundant of all PUFAs, and T2D, with a 20% lower incidence per SD higher LA level. A prevailing concern relates to LA being the metabolic precursor to proinflammatory metabolites, particularly AA-derived eicosanoids, though the deleterious effects are not definitively proven and may depend on a complex interplay of metabolic factors [15,38]. Our observation in EPIC-InterAct that AA (20:4n6) is not associated—but intermediate metabolites GLA (18:3n6), DGLA (20:3n6), and estimated Δ6 desaturase activity are adversely (positively) associated—is in line with a previous Mendelian randomisation study suggesting a potential causal adverse association of estimated Δ6 desaturase activity (desaturation of LA→ GLA) with T2D, but not for Δ5 desaturase activity (DGLA→AA) [9]. Despite the causal inference for estimated Δ6 desaturase activity on T2D risk, the biological mechanisms are speculated but not yet proven [9].

There is little other evidence to compare the EPIC-InterAct findings of the three relatively low-abundance n-6 PUFAs that were associated with lower risk (EDA, 20:2n6) or higher risk (DTA, 22:4n6, and n6-DPA, 22:5n6) of T2D.

### Strengths and Limitations

Key strengths of EPIC-InterAct are its large sample size, prospective study design, and long follow-up. Moreover, the inclusion of EPIC-InterAct populations from eight European countries with diverse dietary intakes, comprehensive T2D case ascertainment and verification, the adjustment for many relevant potential confounders, and a series of sensitivity analyses enabled us to report robust findings, though we cannot rule out residual confounding. Our meta-analysis of other available studies enabled us to provide a systematic comparison with EPIC-InterAct findings and to place them in context of the wider evidence. We did not seek to provide pooled summary estimates that included findings from EPIC-InterAct as its large size with *n* = 12,132 T2D cases far outweighs the incident cases contributed by the other studies (ranging between 71 to 2,499 cases).

Several limitations merit attention. Our measurements of PUFAs might have involved misclassification because of measurement only at baseline and long-term storage of plasma samples. However, samples stored at −196°C are likely to be stable over time [22], and any errors were likely to be at random with respect to case status. We could only examine relative and not absolute concentrations of fatty acids, but this is a valid approach adopted commonly in epidemiological research and tends to provide better interpretation of metabolic inter-relationships [22]. Plasma phospholipid PUFAs may reflect both dietary and metabolic influences, but we could not distinguish these. We were unable to examine correlations between dietary and circulating individual PUFAs since in EPIC-InterAct we did not have dietary data on individual PUFAs. However, in general the correlations with circulating levels are low to modest because of measurement error issues of self-report and the linkage with food composition databases, as well as the different time frames of assessment between recall of habitual intake in a food frequency questionnaire (FFQ) versus circulating blood fatty acids. In contrast, there is evidence from dietary intervention trials that essential PUFAs in the blood (n-3 PUFA ALA, and n-6 PUFA LA) reflect dietary intakes of PUFAs [22]. However, we also need to note that circulating PUFAs may be exchanged across tissues and metabolised to diverse molecules [28]. Our clinical case ascertainment could be limited by potential misclassification due to false positive or negative diagnoses. However, we minimised false positives by applying rigorous verification criteria, while potential false negatives due to undiagnosed incident diabetes can be assumed to be nondifferential with regard to the exposure and hence any potential bias would be unlikely to alter our conclusions based on a relative scale (HR) in our analysis. Being based in European populations, our findings may have limited generalisability to other populations. Our comparative meta-analysis may be limited by using risk estimates per study-specific SD because the biological meaning of one SD may vary by studies. Thus, the estimates should be interpreted cautiously as risks per relative ranking on average, though the per-SD estimates are also advantageous given differences in lipid fractions, the number of fatty acids assessed, and laboratory methods. These differences in PUFA measurements across different cohorts point to the advantage of the standardised laboratory methods for measuring PUFA in EPIC-InterAct.

### Implications

The lack of association of circulating EPA and DHA with T2D in EPIC-InterAct differs from the evidence for their inverse association with cardiovascular disease [39]. Whether or not dietary long-chain n-3 PUFAs reduce cardiovascular events is currently unresolved [39,40], but their overall beneficial effects on lipids (e.g., lowering of triglycerides), together with proposed anti-inflammatory, antiarrhythmic, and antithrombotic effects, and effects on improved endothelial function and blood pressure are plausible mechanisms [26]. In contrast, our current report of a lack of association between circulating EPA or DHA and incident T2D is supported by findings from randomised trials that found no evidence of net benefit or harm of long-chain n-3 supplementation on glycaemia or insulin sensitivity [2,3,26]. Therefore, our current findings advance scientific understanding of the possible differential links between circulating n-3 fatty acids and different health endpoints. Moreover, our findings for DPA and for ALA, both being inversely associated with incident T2D, deserve further follow-up to understand mechanisms and causality.

There has been an ongoing debate about the pros and cons of a diet high in the n6-PUFA LA (18:2n6), mainly for cardiovascular health [14,39,41]. This is important to resolve as LA is ubiquitous, being present in a wide variety of foods [38]. The strong evidence that dietary LA influences circulating LA has been reviewed previously [22], and in that context our finding of the inverse association between circulating LA and T2D incidence may have potential implications for the development of dietary advice. However, future work is warranted to build a body of evidence using diverse study designs that compare dietary LA, circulating LA, and their metabolites for the prevention of T2D.

### Conclusion

We provide robust large-scale evidence that circulating plasma phospholipid n-3 PUFA ALA and n-6 PUFA LA concentrations are associated with lower incidence of T2D. In contrast four n-6 PUFAs (GLA, DGLA, DTA, and n-6DPA) are associated with higher T2D incidence in EPIC-InterAct. These findings (i) highlight that it is important to consider individual PUFAs rather than focusing on overall circulating n-3 or n-6 PUFA groups; (ii) provide robust evidence that circulating LA, the most abundant PUFA, is inversely associated with T2D; and (iii) meaningfully advance our understanding that some fatty acids (EPA and DHA) may be related differently with T2D or cardiovascular disease, while stimulating a scientific debate about the potential role of other, previously less well studied individual fatty acids.

## Supporting Information

S1 Fig Forest plots showing the country-specific HRs and 95% CIs for the association between per 1 SD of (A) total and individual n-3 PUFAs (ALA, EPA, DPA, and DHA) and T2D and (B) total and individual n-6 PUFAs (LA, GLA, EDA, DGLA, AA, DTA, and n6-DPA), as well as (C) fatty acid ratios and T2D from random-effects meta-analysis across the eight countries of EPIC-InterAct. (PDF)

S2 Fig Flow diagram of systematic literature search and identification of studies for the prospective associations of circulating concentrations of n-3 and n-6 PUFAs with incident T2D. (PDF)

S3 Fig Funnel plot for the prospective association of 20:3n6/18:2n6 with incident T2D. (PDF)

S1 PRISMA Preferred Reporting Items for Systematic Reviews and Meta-analyses (PRISMA) checklist (DOC)

S1 STROBE Strengthening the Reporting of Observational Studies in Epidemiology (STROBE) checklist. (DOC)

S1 Table The distribution of individual and total PUFAs in the study subcohort by age, sex, BMI, and by case or noncase status—EPIC-InterAct study. (DOC)

S2 Table The distribution of individual and total PUFAs in the study subcohort by country—EPIC-InterAct study. (DOC)

S3 Table Sensitivity analyses for the association between individual and total PUFAs and fatty acid ratios and incident T2D: EPIC-InterAct Study. (DOC)

S4 Table Pooled HR and 95% CI for associations between quintiles of individual and total PUFAs and fatty acid ratios and incident T2D: EPIC-InterAct Study. (DOC)

S5 Table Study characteristics of prospective studies examining the association of n-3 and n-6 PUFAs with incident T2D. (DOC)

S6 Table Meta-analysis results by lipid fraction for the prospective associations of circulating fatty acids with incidence of T2D. (DOC)

S7 Table Associations of plasma phospholipid n-3 and n-6 PUFAs with incidence of T2D for Models 1, 2 and 3. (DOC)

S1 Text Methods for systematic review and meta-analysis of published literature. (DOC)

S2 Text Analysis plan (August 2014). (PDF)

## Abbreviations

AA: arachidonic acid
ALA: α-linolenic acid
BMI: body mass index
DGLA: dihomo-γ-linolenic acid
DHA: docosahexaenoic acid
DTA: docosatetraenoic acid
EDA: eicosadienoic acid
EPA: eicosapentaenoic acid
EPIC: European Prospective Investigation into Cancer and Nutrition
FAME: fatty acid methyl esters
FFQ: food frequency questionnaire
GLA: γ-linolenic acid
IGF-1: insulin-like growth factor-1
LA: linoleic acid
HR: hazard ratio
MRC: Medical Research Council
n6-DPA: docosapentaenoic acid
PRISMA: Preferred Reporting Items for Systematic Reviews and Meta-analyses
PUFA: polyunsaturated fatty acid
SD: standard deviation
STROBE: Strengthening the Reporting of Observational Studies in Epidemiology
T2D: type 2 diabetes
