# Supplementary material for: Association of Plasma Phospholipid n-3 and n-6 Polyunsaturated Fatty Acids with Type 2 Diabetes: The EPIC-InterAct Case-Cohort Study
Source: PLoS Med. 2016 Jul 19;13(7):e1002094. doi: 10.1371/journal.pmed.1002094 (PMC4951144; doi:10.1371/journal.pmed.1002094)

S1 Fig. Forest plots showing the country-specific HRs and 95% CIs for the association between per 1 SD of (A) total and individual n-3 PUFAs (ALA, EPA, DPA, and DHA) and T2D and (B) total and individual n-6 PUFAs (LA, GLA, EDA, DGLA, AA, DTA, and n6-DPA), as well as (C) fatty acid ratios and T2D from random-effects meta-analysis across the eight countries of EPIC-InterAct.

S1A Fig. (n-3 PUFA - InterAct)

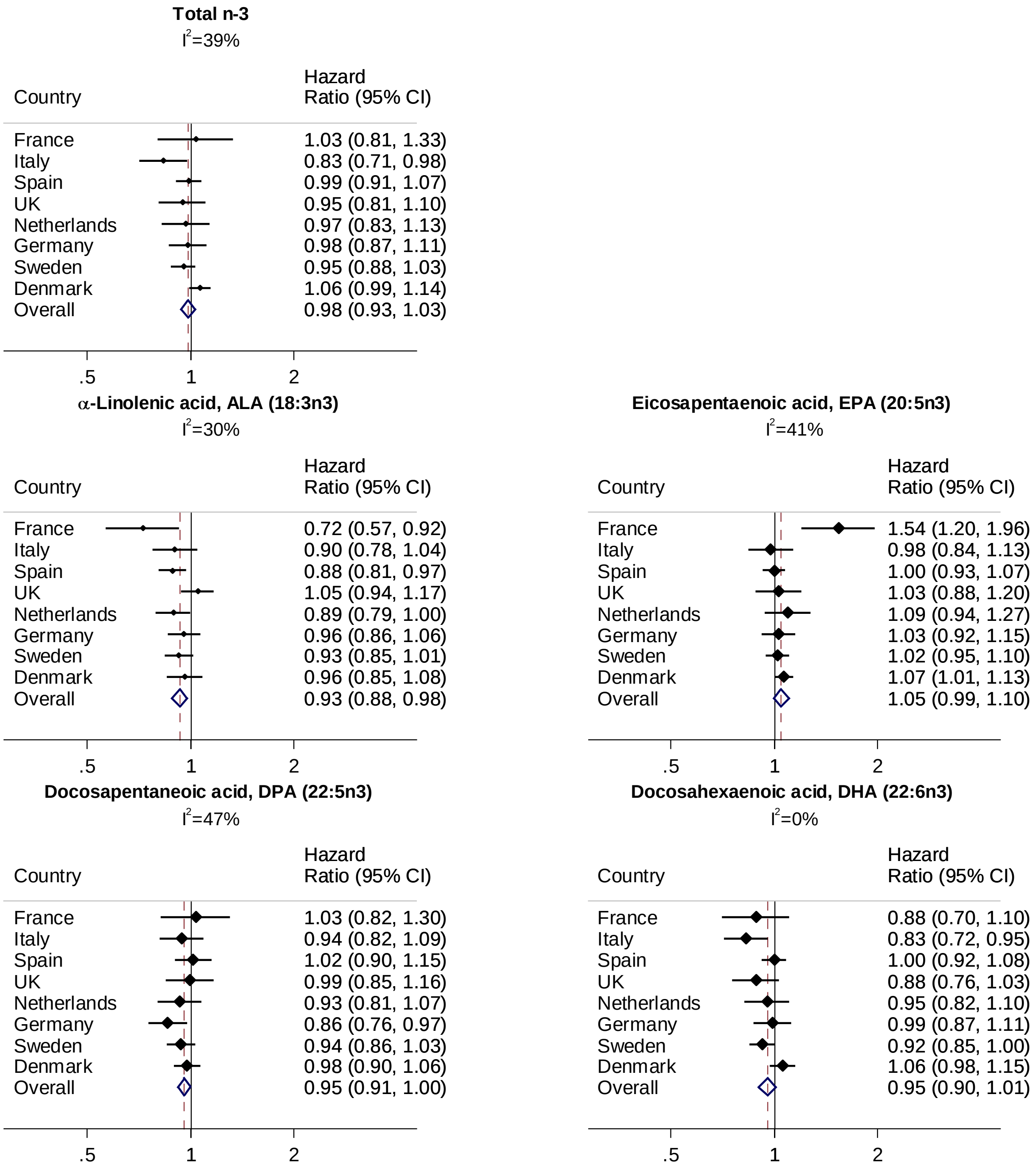

S1B Fig (n-6 PUFA - InterAct)

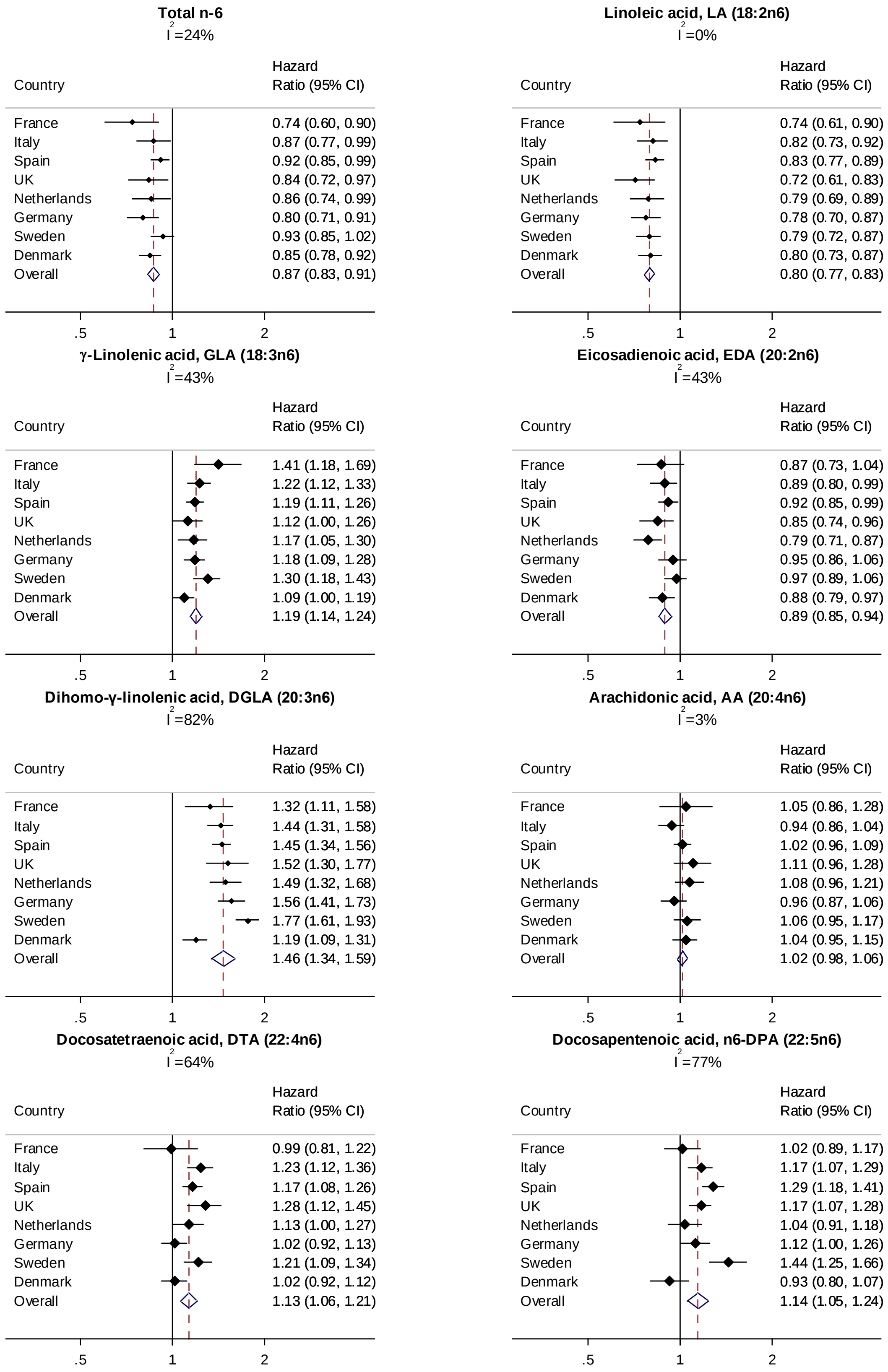

S1C Fig (PUFA ratios - InterAct)

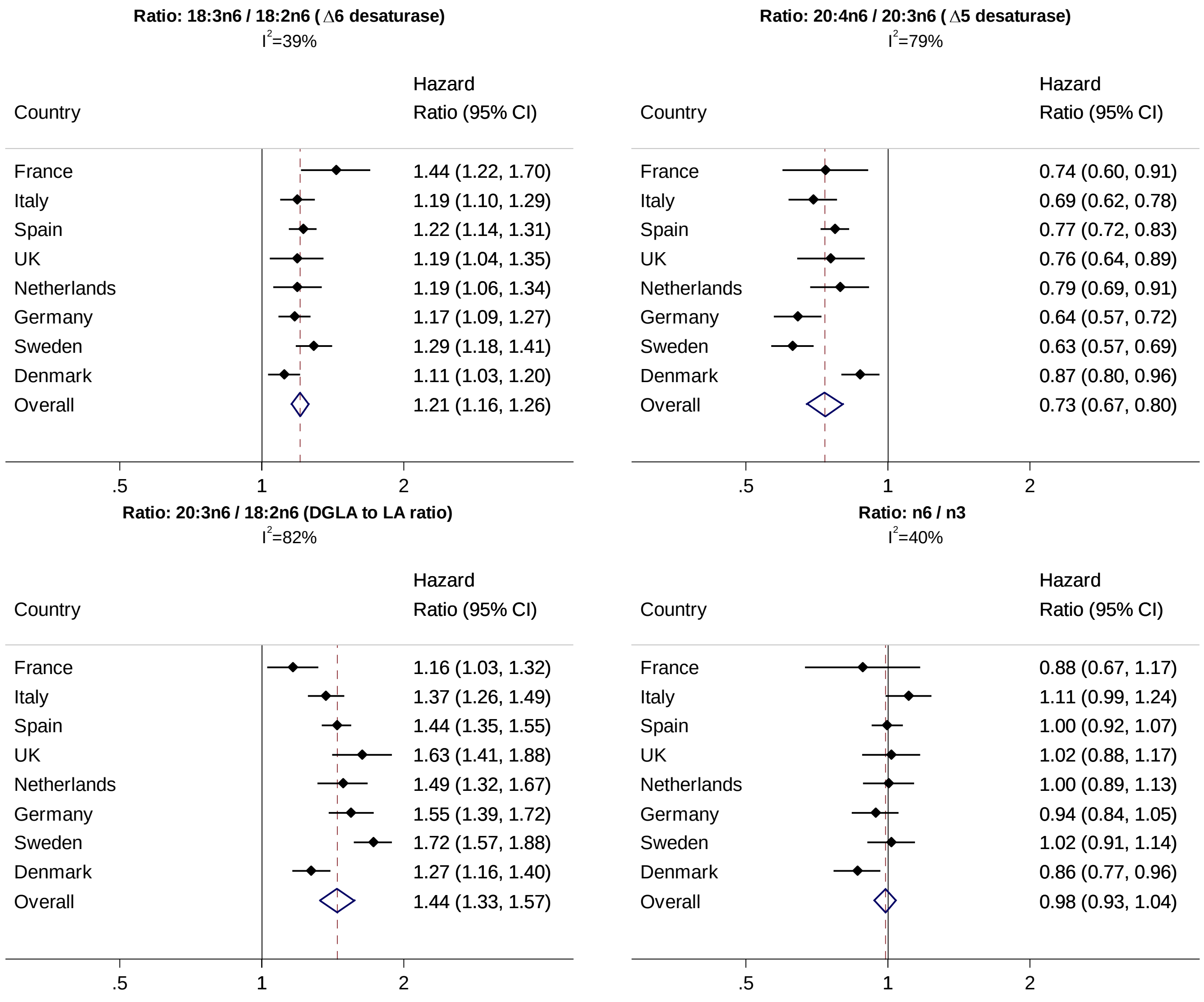

Supplement: S1 Fig — (PDF) [file pmed.1002094.s001.pdf]
