# Supplementary material for: Association of Plasma Phospholipid n-3 and n-6 Polyunsaturated Fatty Acids with Type 2 Diabetes: The EPIC-InterAct Case-Cohort Study
Source: PLoS Med. 2016 Jul 19;13(7):e1002094. doi: 10.1371/journal.pmed.1002094 (PMC4951144; doi:10.1371/journal.pmed.1002094)

S2 Fig. Flow diagram of systematic literature search and identification of studies for the prospective associations of circulating concentrations of n-3 and n-6 PUFAs with incident T2D.

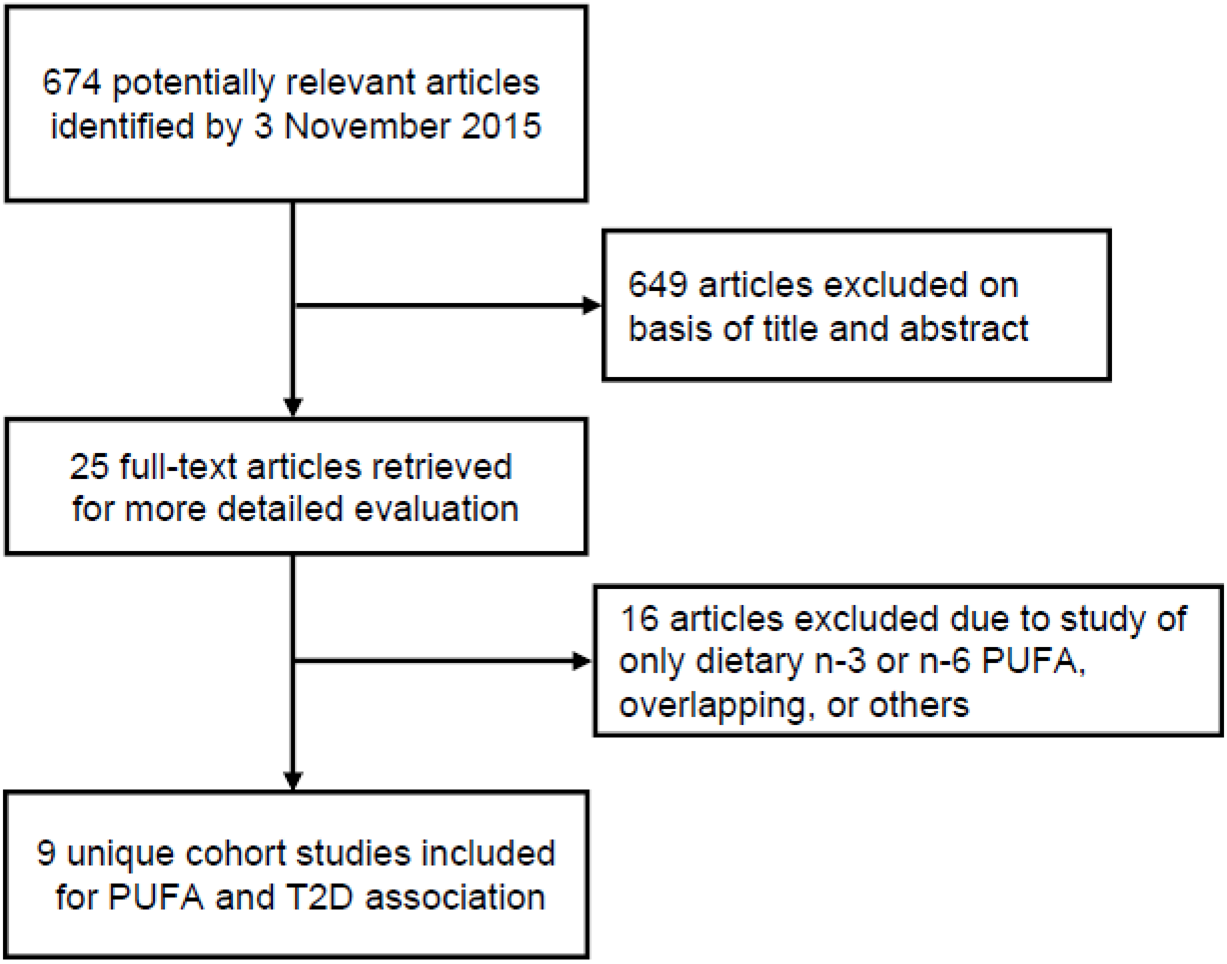

Supplement: S2 Fig — (PDF) [file pmed.1002094.s002.pdf]
