# Supplementary material for: Association of Plasma Phospholipid n-3 and n-6 Polyunsaturated Fatty Acids with Type 2 Diabetes: The EPIC-InterAct Case-Cohort Study
Source: PLoS Med. 2016 Jul 19;13(7):e1002094. doi: 10.1371/journal.pmed.1002094 (PMC4951144; doi:10.1371/journal.pmed.1002094)
Supplement: S3 Fig — (PDF) [file pmed.1002094.s003.pdf]

S3 Fig. Funnel plot for the prospective association of 20:3n6/18:2n6 with incident T2D.

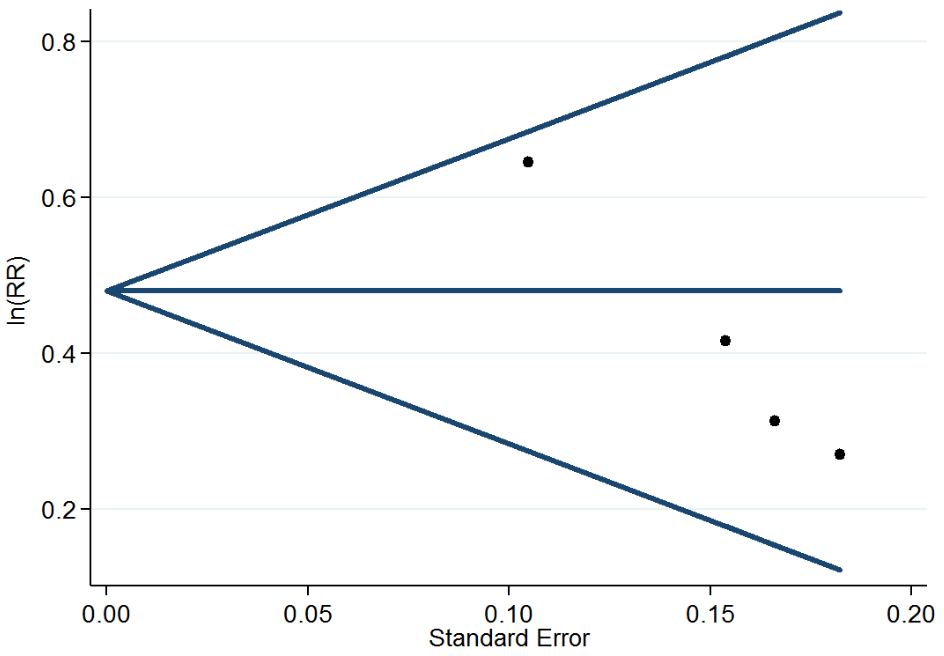

This funnel plot indicates potential publication bias due to the observations depending on standard errors (horizontal axis). Blue lines indicate the summary estimate (horizontal line) and its confidence intervals (non-horizontal lines) according to standard errors. Four dots represent estimates from four single studies (METSIM, Finland; MCCS, Australia; VIP, Sweden; EPIC Norfolk, UK) and were evaluated by a linear regression: $\ln(RR_i)$ = intercept + slope × standard $error_i$. The slope compared to a flat line (Egger's test) and the intercept compared to the point estimate (Begg's test) were both significant, indicating publication bias. The exclusion of the outlying estimate with the highest point estimate did not support a null association, shifting relative risk of 1.62 (95% confidence interval: 1.41, 1.86) to 1.41 (1.17, 1.86). Thus, publication bias was present, but not remarkable enough to alter the inference of positive association
