# Supplementary material for: Association of Plasma Phospholipid n-3 and n-6 Polyunsaturated Fatty Acids with Type 2 Diabetes: The EPIC-InterAct Case-Cohort Study
Source: PLoS Med. 2016 Jul 19;13(7):e1002094. doi: 10.1371/journal.pmed.1002094 (PMC4951144; doi:10.1371/journal.pmed.1002094)
Supplement: S1 PRISMA — (DOC) [file pmed.1002094.s004.doc]

| **Section/topic** | **#** | **Checklist item** | **Reported** |
| --- | --- | --- | --- |
| **TITLE** | | |  |
| Title | 1 | Identify the report as a systematic review, meta-analysis, or both. | The title does not include the specific term of meta-analysis or review, because the focus of the article was on results from the EPIC-InterAct Study, and we made it clear in the manuscript that the meta-analysis was for comparative purposes and that it did not include results from the EPIC-InterAct Study (Methods section, paragraph 7; Discussion section, paragraph 7). The systematic review and meta-analysis were included as a secondary objective (Introduction section, paragraph 3). |
| **ABSTRACT** | | |  |
| Structured summary | 2 | Provide a structured summary including, as applicable: background; objectives; data sources; study eligibility criteria, participants, and interventions; study appraisal and synthesis methods; results; limitations; conclusions and implications of key findings; systematic review registration number. | The abstract does not include this series of information, but includes more brief information because the focus of the article was on results from the EPIC-InterAct Study, and we made it clear in the manuscript that the meta-analysis was for comparative purposes and that it did not include results from the EPIC-InterAct Study (Methods section, paragraph 7; Discussion section, paragraph 7). The systematic review and meta-analysis were included as a secondary part (Introduction section, paragraph 3). |
| **INTRODUCTION** | | |  |
| Rationale | 3 | Describe the rationale for the review in the context of what is already known. | In the second paragraph, a lack of research is noted. In the end of the Introduction, we provided the aim of conducting the review: “To consider the totality of existing evidence, we also conducted a systematic literature review and performed a meta-analysis to compare with the findings from EPIC-InterAct.” This is stated as a secondary objective. |
| Objectives | 4 | Provide an explicit statement of questions being addressed with reference to participants, interventions, comparisons, outcomes, and study design (PICOS). | In the last paragraph of the introduction, the exposure and the outcome are clearly documented. The information on ‘participants’, i.e. adults in the general population as part of the EPIC-InterAct Study and as comparison with published literature is specified, and the entire Introduction part covers this as the risk of developing the outcome of type 2 diabetes. |
| **METHODS** | | |  |
| Protocol and registration | 5 | Indicate if a review protocol exists, if and where it can be accessed (e.g., Web address), and, if available, provide registration information including registration number. | A review protocol was prepared and its availability upon request is indicated in the first paragraph of the Supplementary Information (S1 Text). |
| Eligibility criteria | 6 | Specify study characteristics (e.g., PICOS, length of follow-up) and report characteristics (e.g., years considered, language, publication status) used as criteria for eligibility, giving rationale. | In the Methods subsection of “*Systematic review and meta-analysis of published studies*” (paragraph 7 of methods section), the eligibility criteria are documented. as “prospective studies published by 3rd November 2015 that reported on the association of circulating n-3 or n-6 PUFAs with T2D using PubMed”. This information and no restriction of language and publication date are provided in the first paragraph of S1 Text, too. |
| Information sources | 7 | Describe all information sources (e.g., databases with dates of coverage, contact with study authors to identify additional studies) in the search and date last searched. | “Pubmed” is documented in the subsection, “*Systematic review and meta-analysis of published studies*” (methods section, Paragraph 7), and the first paragraph of the S1 Text. |
| Search | 8 | Present full electronic search strategy for at least one database, including any limits used, such that it could be repeated. | Documented in the first paragraph of the S1 Text. |
| Study selection | 9 | State the process for selecting studies (i.e., screening, eligibility, included in systematic review, and, if applicable, included in the meta-analysis). | S2 Fig documents the process. |
| Data collection process | 10 | Describe method of data extraction from reports (e.g., piloted forms, independently, in duplicate) and any processes for obtaining and confirming data from investigators. | Documented in the first paragraph of the S1 Text, as “For each contributing study, information was extracted according to a pre-specified protocol by two authors independently.” |
| Data items | 11 | List and define all variables for which data were sought (e.g., PICOS, funding sources) and any assumptions and simplifications made. | S5 Table clarifies the information extracted. |
| Risk of bias in individual studies | 12 | Describe methods used for assessing risk of bias of individual studies (including specification of whether this was done at the study or outcome level), and how this information is to be used in any data synthesis. | The risk of bias of each study was not individually examined, because the quality of existing studies on tissue fatty acids and diabetes was identified to be reasonable in the existing review on omega-3 polyunsaturated fatty acids (Ref #6) and because the purpose of this review was to compare between the results from EPIC InterAct and the existing evidence as supplemental information (secondary objective). The focus of the article was on results from the EPIC-InterAct Study, and we made it clear in the manuscript that the meta-analysis was for comparative purposes and that it did not include results from the EPIC-InterAct Study (Methods section, paragraph 7; Discussion section, paragraph 7). |
| Summary measures | 13 | State the principal summary measures (e.g., risk ratio, difference in means). | The end of S1 Text documents use of odds ratios, risk ratios or hazard ratios from each study as measures of the association of interest. |
| Synthesis of results | 14 | Describe the methods of handling data and combining results of studies, if done, including measures of consistency (e.g., I^2^) for each meta-analysis. | The Methods subsection (paragraph 7), *Systematic review and meta-analysis of published studies*, documents dose-response meta-analysis to combine study-specific estimates and to analyse heterogeneity (in the second and third last sentences). |
| Risk of bias across studies | 15 | Specify any assessment of risk of bias that may affect the cumulative evidence (e.g., publication bias, selective reporting within studies). | The Methods subsection (paragraph 7), *Systematic review and meta-analysis of published studies*, documents investigation of publication bias (last sentence). |
| Additional analyses | 16 | Describe methods of additional analyses (e.g., sensitivity or subgroup analyses, meta-regression), if done, indicating which were pre-specified. | Meta-analysis by lipid fraction is documented at the end of the Methods section (paragraph 7). |
| **RESULTS** | | |  |
| Study selection | 17 | Give numbers of studies screened, assessed for eligibility, and included in the review, with reasons for exclusions at each stage, ideally with a flow diagram. | S2 Fig clarifies these statistics. |
| Study characteristics | 18 | For each study, present characteristics for which data were extracted (e.g., study size, PICOS, follow-up period) and provide the citations. | S5 Table clarifies this information. |
| Risk of bias within studies | 19 | Present data on risk of bias of each study and, if available, any outcome level assessment (see item 12). | The information was not specifically assessed in this study. The risk of bias of each study was not individually examined because the quality of existing studies on tissue fatty acids and diabetes was identified to be reasonable in the existing review on omega-3 polyunsaturated fatty acids (Ref #6) and because the purpose of this review was to compare between the results from EPIC InterAct and the existing evidence as supplemental information (secondary objective). The focus of the article was on results from the EPIC-InterAct Study, and we made it clear in the manuscript that the meta-analysis was for comparative purposes and that it did not include results from the EPIC-InterAct Study (Methods section, paragraph 7; Discussion section, paragraph 7). |
| Results of individual studies | 20 | For all outcomes considered (benefits or harms), present, for each study: (a) simple summary data for each intervention group (b) effect estimates and confidence intervals, ideally with a forest plot. | As the meta-analysis was not a primary focus of this manuscript, for simplicity of the presentation for 17 exposure variables (S6 Table), each estimate from each study is not presented, but only synthesised results are presented (main Table 2; S6 Table) |
| Synthesis of results | 21 | Present results of each meta-analysis done, including confidence intervals and measures of consistency. | Presented in Table 2 and S6 Table. |
| Risk of bias across studies | 22 | Present results of any assessment of risk of bias across studies (see Item 15). | S3 Fig presents the publication bias indicated in sensitivity analysis. |
| Additional analysis | 23 | Give results of additional analyses, if done (e.g., sensitivity or subgroup analyses, meta-regression [see Item 16]). | S6 Table indicates the results of meta-analyses by lipid fraction for each fatty acid exposure. |
| **DISCUSSION** | | |  |
| Summary of evidence | 24 | Summarize the main findings including the strength of evidence for each main outcome; consider their relevance to key groups (e.g., healthcare providers, users, and policy makers). | The first paragraph of the Discussion, and the subsections of Discussion section [*Findings in context: n-3 fatty acids* *(Discussion paragraphs 2-4)* and *Findings in context: n-6 fatty acids (Discussion paragraphs 5-6)*] |
| Limitations | 25 | Discuss limitations at study and outcome level (e.g., risk of bias), and at review-level (e.g., incomplete retrieval of identified research, reporting bias). | For the primary findings from the EPIC-InterAct Study, the study-level limitations are documented in paragraph 8 of the discussion). Limitations of the meta-analysis are documented at the end of *Strengths and limitations* of the Discussion section (paragraph 8). |
| Conclusions | 26 | Provide a general interpretation of the results in the context of other evidence, and implications for future research. | Along with the findings from EPIC-InterAct, the implications are documented in the *Implications* subsection in the Discussion (paragraph 9). |
| **FUNDING** | | |  |
| Funding | 27 | Describe sources of funding for the systematic review and other support (e.g., supply of data); role of funders for the systematic review. | Documented within the submission process at PLOS Medicine in the Financial Disclosure field. |
