# Supplementary material for: Association of Plasma Phospholipid n-3 and n-6 Polyunsaturated Fatty Acids with Type 2 Diabetes: The EPIC-InterAct Case-Cohort Study
Source: PLoS Med. 2016 Jul 19;13(7):e1002094. doi: 10.1371/journal.pmed.1002094 (PMC4951144; doi:10.1371/journal.pmed.1002094)
Supplement: S1 Table — (DOC) [file pmed.1002094.s006.doc]

**S1 Table. The distribution of individual and total PUFAs in the study subcohort by age, sex, BMI, and by case or noncase status—EPIC-InterAct study**

|  | **Sub-cohort (N=15919)** | | | | | | | | | | | | | | | | | | **T2D cases** | |
| --- | --- | --- | --- | --- | --- | --- | --- | --- | --- | --- | --- | --- | --- | --- | --- | --- | --- | --- | --- | --- |
|  | **Age (years)** | | | | | | **Sex** | | | | **BMI (kg/m^2^)** | | | | | | **Non-cases** | |
|  | **<40** | | **40-<60** | | **≥60** | | **Men** | | **Women** | | **<25** | | **25-<30** | | **≥30** | |
|  | **N=1524** | | **N=10919** | | **N=3476** | | **N=6002** | | **N=9917** | | **N=7052** | | **N=6255** | | **N=2502** | | **N=15164** | | **N=12132** | |
|  | **mean** | **sd** | **mean** | **sd** | **mean** | **sd** | **mean** | **sd** | **mean** | **sd** | **mean** | **sd** | **mean** | **sd** | **mean** | **sd** | **mean** | **sd** | **mean** | **sd** |
| **Polyunsaturated fatty acids** | 43.28 | 1.95 | 42.70 | 2.12 | 42.21 | 2.16 | 42.46 | 2.18 | 42.76 | 2.10 | 42.64 | 2.07 | 42.64 | 2.16 | 42.67 | 2.25 | 43.19 | 1.98 | 42.52 | 2.14 |
| **n-3** | 5.90 | 1.47 | 6.68 | 1.91 | 7.26 | 2.15 | 6.86 | 2.00 | 6.66 | 1.94 | 6.75 | 2.03 | 6.76 | 1.96 | 6.63 | 1.80 | 5.93 | 1.47 | 6.68 | 1.92 |
| α-Linolenic acid, ALA (18:3n3) | 0.32 | 0.18 | 0.30 | 0.16 | 0.32 | 0.18 | 0.30 | 0.17 | 0.31 | 0.17 | 0.33 | 0.18 | 0.30 | 0.16 | 0.28 | 0.16 | 0.32 | 0.18 | 0.30 | 0.16 |
| Eicosapentaenoic acid, EPA (20:5n3), | 0.90 | 0.52 | 1.23 | 0.79 | 1.44 | 0.90 | 1.33 | 0.84 | 1.19 | 0.78 | 1.24 | 0.81 | 1.26 | 0.83 | 1.22 | 0.77 | 0.92 | 0.52 | 1.26 | 0.80 |
| Docosapentaneoic acid, DPA (22:5n3) | 0.81 | 0.24 | 0.89 | 0.24 | 0.97 | 0.23 | 0.93 | 0.24 | 0.88 | 0.23 | 0.93 | 0.23 | 0.89 | 0.24 | 0.82 | 0.23 | 0.82 | 0.24 | 0.88 | 0.23 |
| Docosahexaenoic acid, DHA (22:6n3) | 3.86 | 1.08 | 4.26 | 1.22 | 4.54 | 1.33 | 4.29 | 1.24 | 4.28 | 1.25 | 4.25 | 1.30 | 4.31 | 1.23 | 4.31 | 1.15 | 3.87 | 1.08 | 4.24 | 1.22 |
|  |  |  |  |  |  |  |  |  |  |  |  |  |  |  |  |  |  |  |  |  |
| **n-6** | 37.39 | 2.46 | 36.02 | 2.87 | 34.94 | 3.03 | 35.60 | 2.98 | 36.10 | 2.90 | 35.89 | 2.93 | 35.89 | 2.96 | 36.04 | 2.96 | 37.27 | 2.50 | 35.84 | 2.89 |
| Linoleic acid, LA (18:2n6) | 23.77 | 3.09 | 22.60 | 3.15 | 22.12 | 3.14 | 22.37 | 3.11 | 22.75 | 3.19 | 23.03 | 3.18 | 22.38 | 3.13 | 21.97 | 3.06 | 23.53 | 3.12 | 22.20 | 3.14 |
| γ-Linolenic acid, GLA (18:3n6) | 0.07 | 0.04 | 0.08 | 0.05 | 0.08 | 0.05 | 0.09 | 0.05 | 0.08 | 0.05 | 0.07 | 0.04 | 0.08 | 0.05 | 0.10 | 0.05 | 0.07 | 0.05 | 0.09 | 0.05 |
| Eicosadienoic acid, EDA (20:2n6) | 0.39 | 0.08 | 0.38 | 0.07 | 0.39 | 0.08 | 0.37 | 0.06 | 0.39 | 0.08 | 0.38 | 0.07 | 0.38 | 0.08 | 0.38 | 0.07 | 0.39 | 0.08 | 0.38 | 0.07 |
| Dihomo-γ-linolenic acid, DGLA (20:3n6) | 3.16 | 0.79 | 3.13 | 0.77 | 3.14 | 0.77 | 3.10 | 0.74 | 3.16 | 0.80 | 2.94 | 0.73 | 3.20 | 0.76 | 3.50 | 0.76 | 3.25 | 0.81 | 3.31 | 0.82 |
| Arachidonic acid, AA (20:4n6) | 9.47 | 1.72 | 9.35 | 1.81 | 8.77 | 1.77 | 9.22 | 1.82 | 9.24 | 1.80 | 8.99 | 1.74 | 9.36 | 1.83 | 9.61 | 1.85 | 9.51 | 1.73 | 9.39 | 1.81 |
| Docosatetraenoic acid, DTA (22:4n6) | 0.30 | 0.07 | 0.29 | 0.08 | 0.27 | 0.08 | 0.29 | 0.08 | 0.28 | 0.08 | 0.28 | 0.08 | 0.29 | 0.08 | 0.29 | 0.08 | 0.30 | 0.07 | 0.29 | 0.08 |
| Docosapentenoic acid, n6-DPA (22:5n6) | 0.22 | 0.09 | 0.19 | 0.08 | 0.18 | 0.09 | 0.18 | 0.08 | 0.20 | 0.09 | 0.19 | 0.09 | 0.19 | 0.08 | 0.19 | 0.08 | 0.22 | 0.09 | 0.19 | 0.08 |
| **Ratios** |  |  |  |  |  |  |  |  |  |  |  |  |  |  |  |  |  |  |  |  |
| 18:3n6/18:2n6 (Δ6 desaturase) | 0.003 | 0.002 | 0.004 | 0.002 | 0.004 | 0.002 | 0.004 | 0.002 | 0.004 | 0.002 | 0.003 | 0.002 | 0.004 | 0.002 | 0.004 | 0.003 | 0.003 | 0.002 | 0.004 | 0.0034 |
| 20:4n6/20:3n6 (Δ5 desaturase) | 3.17 | 0.96 | 3.15 | 0.95 | 2.94 | 0.86 | 3.13 | 0.91 | 3.10 | 0.95 | 3.21 | 0.92 | 3.08 | 0.95 | 2.88 | 0.91 | 3.11 | 0.97 | 3.01 | 0.93 |
| 20:3n6/18:2n6 (DGLA to LA ratio) | 0.14 | 0.05 | 0.14 | 0.05 | 0.15 | 0.05 | 0.14 | 0.04 | 0.14 | 0.05 | 0.13 | 0.04 | 0.15 | 0.05 | 0.16 | 0.05 | 0.14 | 0.05 | 0.15 | 0.05 |
| n6: n3 | 6.80 | 2.01 | 5.89 | 1.93 | 5.31 | 1.89 | 5.70 | 1.93 | 5.94 | 1.99 | 5.86 | 2.06 | 5.81 | 1.90 | 5.89 | 1.87 | 6.74 | 1.98 | 5.86 | 1.91 |

T2D = type 2 diabetes; total sub-cohort with n=15,919 includes n=755 T2D cases in sub-cohort as per the design of a case-cohort study. (N=15,164 non-cases represent the sub-cohort minus the 755 incident T2D cases in the sub-cohort; this is to enable a comparison of non-cases with T2D cases).
