# Supplementary material for: Association of Plasma Phospholipid n-3 and n-6 Polyunsaturated Fatty Acids with Type 2 Diabetes: The EPIC-InterAct Case-Cohort Study
Source: PLoS Med. 2016 Jul 19;13(7):e1002094. doi: 10.1371/journal.pmed.1002094 (PMC4951144; doi:10.1371/journal.pmed.1002094)
Supplement: S2 Table — (DOC) [file pmed.1002094.s007.doc]

|  |  | **S2 Table. The distribution of individual and total PUFAs in the study subcohort by country—EPIC-InterAct study** | | | | | | | | | | | | | | | | | | |
| --- | --- | --- | --- | --- | --- | --- | --- | --- | --- | --- | --- | --- | --- | --- | --- | --- | --- | --- | --- | --- |
|  | | | **France**  **N=587** | | **Italy** | | **Spain** | | **UK** | | **Netherlands** | | **Germany** | | **Sweden** | | **Denmark** | | **Total**  **15919** | |
|  | | |  |  | **N=2006** | | **N=3555** | | **N=1310** | | **N=1500** | | **N=2045** | | **N=2826** | | **N=2090** | |  |  |
|  | | | **mean** | **sd** | **mean** | **sd** | **mean** | **sd** | **mean** | **sd** | **mean** | **sd** | **mean** | **sd** | **mean** | **sd** | **mean** | **sd** | **mean** | **sd** |
| **Polyunsaturated fatty acids** | | | 42.30 | 2.19 | 42.08 | 1.91 | 43.55 | 2.18 | 42.40 | 2.22 | 43.14 | 2.04 | 43.18 | 1.87 | 41.79 | 1.86 | 42.20 | 2.03 | 42.65 | 2.13 |
|  | | |  |  |  |  |  |  |  |  |  |  |  |  |  |  |  |  |  |  |
| **n-3** | | | 7.36 | 1.77 | 5.47 | 1.34 | 6.61 | 1.66 | 6.85 | 2.08 | 5.77 | 1.56 | 6.15 | 1.53 | 7.57 | 1.91 | 8.02 | 2.26 | 6.73 | 1.96 |
| α-Linolenic acid, ALA (18:3n3) | | | 0.34 | 0.22 | 0.30 | 0.13 | 0.23 | 0.14 | 0.38 | 0.23 | 0.32 | 0.21 | 0.35 | 0.17 | 0.35 | 0.15 | 0.30 | 0.11 | 0.31 | 0.17 |
| Eicosapentaenoic acid, EPA (20:5n3) | | | 1.21 | 0.63 | 0.80 | 0.46 | 1.04 | 0.71 | 1.29 | 0.79 | 0.97 | 0.58 | 1.14 | 0.63 | 1.56 | 0.80 | 1.87 | 1.02 | 1.24 | 0.81 |
| Docosapentaneoic acid, DPA (22:5n3) | | | 1.00 | 0.21 | 0.77 | 0.16 | 0.67 | 0.14 | 1.02 | 0.22 | 0.96 | 0.21 | 0.92 | 0.19 | 1.03 | 0.20 | 1.05 | 0.21 | 0.90 | 0.24 |
| Docosahexaenoic acid, DHA (22:6n3) | | | 4.81 | 1.19 | 3.61 | 0.96 | 4.68 | 1.07 | 4.16 | 1.35 | 3.52 | 1.07 | 3.75 | 0.99 | 4.64 | 1.25 | 4.79 | 1.30 | 4.28 | 1.25 |
|  | | |  |  |  |  |  |  |  |  |  |  |  |  |  |  |  |  |  |  |
| **n-6** | | | 34.94 | 3.00 | 36.61 | 2.25 | 36.94 | 2.89 | 35.55 | 3.09 | 37.37 | 2.68 | 37.03 | 2.39 | 34.21 | 2.39 | 34.18 | 2.77 | 35.91 | 2.94 |
| Linoleic acid, LA (18:2n6c) | | | 21.06 | 3.41 | 21.51 | 2.77 | 23.18 | 3.31 | 23.03 | 3.30 | 23.76 | 3.24 | 23.27 | 3.00 | 22.12 | 2.75 | 22.02 | 3.04 | 22.61 | 3.17 |
| γ-Linolenic acid, GLA (18:3n6) | | | 0.06 | 0.05 | 0.10 | 0.05 | 0.08 | 0.04 | 0.08 | 0.05 | 0.08 | 0.05 | 0.09 | 0.05 | 0.07 | 0.04 | 0.08 | 0.04 | 0.08 | 0.05 |
| Eicosadienoic acid, EDA (20:2n6) | | | 0.39 | 0.08 | 0.37 | 0.07 | 0.37 | 0.06 | 0.40 | 0.08 | 0.41 | 0.10 | 0.39 | 0.07 | 0.38 | 0.06 | 0.35 | 0.06 | 0.38 | 0.07 |
| Dihomo-γ-linolenic acid, DGLA (20:3n6) | | | 3.15 | 0.86 | 3.70 | 0.77 | 3.06 | 0.76 | 3.19 | 0.75 | 3.30 | 0.75 | 3.15 | 0.71 | 2.94 | 0.68 | 2.83 | 0.68 | 3.14 | 0.78 |
| Arachidonic acid, AA (20:4n6) | | | 9.73 | 1.70 | 10.31 | 1.79 | 9.79 | 1.78 | 8.40 | 1.70 | 9.28 | 1.74 | 9.61 | 1.70 | 8.30 | 1.41 | 8.51 | 1.49 | 9.24 | 1.81 |
| Docosatetraenoic acid, DTA (22:4n6) | | | 0.31 | 0.07 | 0.34 | 0.08 | 0.27 | 0.07 | 0.28 | 0.08 | 0.32 | 0.09 | 0.31 | 0.07 | 0.26 | 0.06 | 0.25 | 0.06 | 0.28 | 0.08 |
| Docosapentenoic acid, n6-DPA (22:5n6) | | | 0.24 | 0.11 | 0.28 | 0.08 | 0.19 | 0.07 | 0.17 | 0.10 | 0.22 | 0.09 | 0.21 | 0.07 | 0.14 | 0.05 | 0.13 | 0.05 | 0.19 | 0.09 |
|  | | |  |  |  |  |  |  |  |  |  |  |  |  |  |  |  |  |  |  |
| **Ratios** | | |  |  |  |  |  |  |  |  |  |  |  |  |  |  |  |  |  |  |
| 18:3n6 /18:2n6 ((Δ6 desaturase) | | | 0.003 | 0.003 | 0.005 | 0.003 | 0.004 | 0.002 | 0.003 | 0.002 | 0.003 | 0.002 | 0.004 | 0.003 | 0.003 | 0.002 | 0.004 | 0.002 | 0.004 | 0.002 |
| 20:4n6 /20:3n6 (Δ5 desaturase) | | | 3.30 | 0.98 | 2.92 | 0.83 | 3.40 | 1.08 | 2.78 | 0.87 | 2.96 | 0.89 | 3.19 | 0.90 | 2.96 | 0.80 | 3.17 | 0.88 | 3.11 | 0.94 |
| 20:3n6 / 18:2n6 (DGLA to LA ratio) | | | 0.16 | 0.07 | 0.18 | 0.05 | 0.14 | 0.04 | 0.14 | 0.05 | 0.14 | 0.04 | 0.14 | 0.04 | 0.14 | 0.04 | 0.13 | 0.04 | 0.14 | 0.05 |
| n6 / n3 | | | 5.10 | 1.60 | 7.10 | 1.83 | 6.00 | 1.79 | 5.80 | 2.37 | 6.99 | 2.10 | 6.42 | 1.75 | 4.83 | 1.36 | 4.63 | 1.46 | 5.85 | 1.97 |
