# Supplementary material for: Association of Plasma Phospholipid n-3 and n-6 Polyunsaturated Fatty Acids with Type 2 Diabetes: The EPIC-InterAct Case-Cohort Study
Source: PLoS Med. 2016 Jul 19;13(7):e1002094. doi: 10.1371/journal.pmed.1002094 (PMC4951144; doi:10.1371/journal.pmed.1002094)
Supplement: S3 Table — (DOC) [file pmed.1002094.s008.doc]

**S3 Table. Sensitivity analyses for the association between individual and total PUFAs and fatty acid ratios and incident T2D: EPIC-InterAct Study**

|  | Model 3 | | Model 3a | | Model 3b | | Model 3c | | Model 3d | | Model 3e | | Model 3f | |
| --- | --- | --- | --- | --- | --- | --- | --- | --- | --- | --- | --- | --- | --- | --- |
|  | HR | 95% CI | HR | 95% CI | HR | 95% CI | HR | 95% CI | HR | 95% CI | HR | 95% CI | HR | 95% CI |
|  |  |  |  |  |  |  |  |  |  |  |  |  |  |  |
| **n-3 PUFA** | 0.98 | 0.93, 1.03 | 0.94 | 0.88, 1.00 | 1.07 | 1.03, 1.11 | 0.98 | 0.93, 1.03 | 0.97 | 0.93, 1.01 | 0.99 | 0.95, 1.04 | 0.98 | 0.95, 1.02 |
| α-Linolenic acid, ALA (18:3n3) | 0.93 | 0.88, 0.98 | 0.95 | 0.89, 1.01 | 0.92 | 0.88, 0.97 | 0.93 | 0.88, 0.98 | 0.9 | 0.84, 0.96 | 0.93 | 0.88, 0.99 | 0.93 | 0.88, 0.98 |
| Eicosapentaenoic acid, EPA (20:5n3) | 1.05 | 0.99, 1.10 | 1.01 | 0.95, 1.07 | 1.09 | 1.04, 1.14 | 1.05 | 0.99, 1.11 | 0.99 | 0.95, 1.03 | 1.04 | 0.98, 1.1 | 1.03 | 1.00, 1.06 |
| Docosapentaneoic acid, DPA (22:5n3) | 0.95 | 0.91, 1.00 | 0.90 | 0.85, 0.94 | 1.00 | 0.95, 1.04 | 0.95 | 0.91, 1.00 | 1.02 | 0.98, 1.07 | 0.97 | 0.93, 1.02 | 0.96 | 0.92, 1.00 |
| Docosahexaenoic acid, DHA (22:6n3) | 0.95 | 0.90, 1.01 | 0.92 | 0.87, 0.98 | 1.05 | 1.00, 1.11 | 0.95 | 0.90, 1.01 | 0.98 | 0.93, 1.03 | 0.98 | 0.94, 1.03 | 0.96 | 0.92, 1.00 |
|  |  |  |  |  |  |  |  |  |  |  |  |  |  |  |
| **n-6 PUFA** | 0.87 | 0.83, 0.91 | 1.07 | 0.99, 1.15 | 0.9 | 0.86, 0.95 | 0.87 | 0.83, 0.91 | 0.88 | 0.85, 0.92 | 0.88 | 0.84, 0.92 | 0.91 | 0.88, 0.94 |
| Linoleic acid, LA (18:2n6) | 0.80 | 0.77, 0.83 | 0.92 | 0.87, 0.97 | 0.84 | 0.81, 0.87 | 0.80 | 0.77, 0.83 | 0.78 | 0.74, 0.82 | 0.80 | 0.77, 0.83 | 0.82 | 0.79, 0.85 |
| γ-Linolenic acid, GLA (18:3n6) | 1.19 | 1.14, 1.24 | 1.16 | 1.08, 1.25 | 1.08 | 1.02, 1.15 | 1.19 | 1.14, 1.24 | 1.20 | 1.16, 1.25 | 1.17 | 1.13, 1.22 | 1.19 | 1.13, 1.25 |
| Eicosadienoic acid, EDA (20:2n6) | 0.89 | 0.85, 0.94 | 0.88 | 0.83, 0.94 | 0.89 | 0.85, 0.93 | 0.89 | 0.85, 0.94 | 0.95 | 0.88, 1.02 | 0.93 | 0.89, 0.98 | 0.91 | 0.87, 0.95 |
| Dihomo-γ-linolenic acid, DGLA (20:3n6) | 1.46 | 1.34, 1.59 | 1.37 | 1.24, 1.51 | 1.37 | 1.24, 1.51 | 1.46 | 1.33, 1.59 | 1.45 | 1.3, 1.62 | 1.47 | 1.34, 1.61 | 1.43 | 1.31, 1.57 |
| Arachidonic acid, AA (20:4n6) | 1.02 | 0.98, 1.06 | 1.04 | 1.00, 1.08 | 1.04 | 1.00, 1.08 | 1.02 | 0.98, 1.05 | 1.06 | 0.99, 1.14 | 1.03 | 0.99, 1.07 | 1.04 | 0.99, 1.09 |
| Docosatetraenoic acid, DTA (22:4n6) | 1.13 | 1.06, 1.21 | 1.08 | 1.00, 1.16 | 1.03 | 0.96, 1.11 | 1.13 | 1.06, 1.20 | 1.23 | 1.15, 1.32 | 1.15 | 1.08, 1.22 | 1.14 | 1.08, 1.21 |
| Docosapentenoic acid, DPA (22:5n6) | 1.14 | 1.05, 1.24 | 1.11 | 1.02, 1.20 | 1.08 | 0.98, 1.18 | 1.14 | 1.05, 1.24 | 1.18 | 1.08, 1.29 | 1.15 | 1.06, 1.24 | 1.14 | 1.06, 1.23 |
| **Ratios** |  |  |  |  |  |  |  |  |  |  |  |  |  |  |
| 18:3n6 / 18:2n6 (Δ6 desaturase) | 1.21 | 1.16, 1.26 | 1.15 | 1.08, 1.23 | 1.10 | 1.04, 1.17 | 1.20 | 1.16, 1.25 | 1.23 | 1.18, 1.27 | 1.19 | 1.15, 1.23 | 1.21 | 1.15, 1.26 |
| 20:4n6 / 20:3n6 (Δ5 desaturase) | 0.73 | 0.67, 0.80 | 0.78 | 0.72, 0.86 | 0.78 | 0.71, 0.85 | 0.73 | 0.67, 0.80 | 0.75 | 0.66, 0.86 | 0.74 | 0.68, 0.80 | 0.74 | 0.67, 0.81 |
| 20:3n6 / 18:2n6 (DGLA to LA ratio) | 1.44 | 1.33, 1.57 | 1.32 | 1.19, 1.45 | 1.36 | 1.24, 1.49 | 1.44 | 1.32, 1.57 | 1.46 | 1.30, 1.65 | 1.45 | 1.33, 1.58 | 1.44 | 1.33, 1.56 |
| n6/n3 | 0.98 | 0.93, 1.04 | 1.07 | 1.00, 1.14 | 0.93 | 0.89, 0.98 | 0.98 | 0.93, 1.04 | 1.00 | 0.96, 1.05 | 0.98 | 0.93, 1.03 | 0.98 | 0.93, 1.04 |

Hazard Ratios (HRs) and confidence intervals (CI) are per 1 SD increase in fatty acid relative concentration.

Model 3: centre, age (as underlying timescale), sex, physical activity, smoking, education level, BMI, total energy intake, alcohol, meat, fruit and vegetables, soft drinks, dairy, fish and shellfish, nuts and seeds, vegetable oil, olive oil, margarine.

Model 3a - Model 3 + c14:0, c16:0, c18:0.

Model 3b - Model 3 + c15:0, c17:0.

Model 3c - Model 3 + prevalent MI, stroke, cancer.

Model 3d – Model 3 + baseline HbA1c.

Model 3e - Model 3 excluding individuals with baseline HbA1c≥6.5%;

Model 3f – Model 3 excluding individuals with incident T2D diagnosed within the first 2 years after baseline.
