# Supplementary material for: Association of Plasma Phospholipid n-3 and n-6 Polyunsaturated Fatty Acids with Type 2 Diabetes: The EPIC-InterAct Case-Cohort Study
Source: PLoS Med. 2016 Jul 19;13(7):e1002094. doi: 10.1371/journal.pmed.1002094 (PMC4951144; doi:10.1371/journal.pmed.1002094)
Supplement: S4 Table — (DOC) [file pmed.1002094.s009.doc]

**S4 Table. Pooled HR and 95% CI for associations between quintiles of individual and total PUFAs and fatty acid ratios and incident T2D: EPIC-InterAct Study**

|  | **Q2 vs Q1** | | | | | | **Q3 vs Q1** | | | | | | **Q4 vs Q1** | | | | | | **Q5 vs Q1** | | | | | |  | |  |
| --- | --- | --- | --- | --- | --- | --- | --- | --- | --- | --- | --- | --- | --- | --- | --- | --- | --- | --- | --- | --- | --- | --- | --- | --- | --- | --- | --- |
| **Model 1** | **HR** | | **Lower** | | **Upper** | | **HR** | | **Lower** | | **Upper** | | **HR** | | **Lower** | | **Upper** | | **HR** | | **Lower** | | **Upper** | | **p-trend** | |  |
|  |  | |  | |  | |  | |  | |  | |  | |  | |  | |  | |  | |  | |  | |  |
|  |  | |  | |  | |  | |  | |  | |  | |  | |  | |  | |  | |  | |  | |  |
| **n-3 PUFA** | 0.96 | | 0.86 | | 1.07 | | 0.94 | | 0.83 | | 1.06 | | 0.94 | | 0.83 | | 1.05 | | 0.87 | | 0.77 | | 0.99 | | 0.040 | |  |
| α-Linolenic acid, ALA (18:3n3) | 0.94 | | 0.81 | | 1.10 | | 0.86 | | 0.75 | | 1.00 | | 0.85 | | 0.72 | | 0.99 | | 0.73 | | 0.62 | | 0.86 | | <0.001 | |  |
| Eicosapentaenoic acid, EPA (20:5n3) | 1.06 | | 0.93 | | 1.20 | | 1.09 | | 0.93 | | 1.28 | | 1.09 | | 0.94 | | 1.27 | | 1.07 | | 0.90 | | 1.26 | | 0.143 | |  |
| Docosapentaneoic acid, DPA (22:5n3) | 0.96 | | 0.81 | | 1.14 | | 0.92 | | 0.77 | | 1.09 | | 0.90 | | 0.68 | | 1.17 | | 0.85 | | 0.67 | | 1.07 | | 0.008 | |  |
| Docosahexaenoic acid, DHA (22:6n3) | 1.07 | | 0.97 | | 1.19 | | 0.97 | | 0.87 | | 1.08 | | 0.93 | | 0.83 | | 1.05 | | 0.92 | | 0.82 | | 1.04 | | 0.021 | |  |
|  |  | |  | |  | |  | |  | |  | |  | |  | |  | |  | |  | |  | |  | |  |
| **n-6 PUFA** | 0.98 | | 0.87 | | 1.10 | | 0.88 | | 0.80 | | 0.98 | | 0.88 | | 0.79 | | 0.98 | | 0.79 | | 0.70 | | 0.90 | | <0.001 | |  |
| Linoleic acid, LA (18:2n6c) | 0.83 | | 0.73 | | 0.94 | | 0.75 | | 0.68 | | 0.84 | | 0.68 | | 0.59 | | 0.77 | | 0.58 | | 0.51 | | 0.67 | | <0.001 | |  |
| γ-Linolenic acid, GLA (18:3n6) | 1.20 | | 1.08 | | 1.33 | | 1.47 | | 1.27 | | 1.70 | | 1.65 | | 1.35 | | 2.01 | | 2.04 | | 1.70 | | 2.45 | | <0.001 | |  |
| Eicosadienoic acid, EDA (20:2n6) | 0.98 | | 0.89 | | 1.08 | | 0.87 | | 0.77 | | 0.97 | | 0.92 | | 0.83 | | 1.01 | | 0.77 | | 0.67 | | 0.89 | | <0.001 | |  |
| Dihomo-γ-linolenic acid, DGLA (20:3n6) | 1.36 | | 1.11 | | 1.67 | | 1.73 | | 1.44 | | 2.06 | | 2.26 | | 1.84 | | 2.77 | | 3.10 | | 2.32 | | 4.14 | | <0.001 | |  |
| Arachidonic acid, AA (20:4n6) | 0.94 | | 0.85 | | 1.04 | | 1.10 | | 0.99 | | 1.22 | | 1.04 | | 0.93 | | 1.16 | | 1.03 | | 0.92 | | 1.17 | | 0.076 | |  |
| Docosatetraenoic acid, DTA (22:4n6) | 1.06 | | 0.90 | | 1.24 | | 1.14 | | 1.03 | | 1.27 | | 1.24 | | 0.99 | | 1.56 | | 1.47 | | 1.19 | | 1.81 | | <0.001 | |  |
| Docosapentenoic acid, n6-DPA (22:5n6) | 1.11 | | 0.87 | | 1.40 | | 1.22 | | 0.97 | | 1.52 | | 1.29 | | 0.99 | | 1.68 | | 1.58 | | 1.03 | | 2.42 | | 0.009 | |  |
| **Ratios** |  | |  | |  | |  | |  | |  | |  | |  | |  | |  | |  | |  | |  | |  |
| 18:3n6 / 18:2n6 (Δ6 desaturase) | 1.23 | | 1.11 | | 1.37 | | 1.54 | | 1.37 | | 1.73 | | 1.84 | | 1.48 | | 2.28 | | 2.18 | | 1.88 | | 2.54 | | <0.001 | |  |
| 20:4n6 / 20:3n6 (Δ5 desaturase) | 0.76 | | 0.68 | | 0.86 | | 0.68 | | 0.62 | | 0.75 | | 0.57 | | 0.51 | | 0.65 | | 0.47 | | 0.39 | | 0.57 | | <0.001 | |  |
| 20:3n6 / 18:2n6 (DGLA to LA ratio) | 1.36 | | 1.18 | | 1.57 | | 1.69 | | 1.50 | | 1.89 | | 2.39 | | 1.94 | | 2.94 | | 3.24 | | 2.47 | | 4.26 | | <0.001 | |  |
| n6/n3 | 1.00 | | 0.91 | | 1.10 | | 1.02 | | 0.92 | | 1.12 | | 1.14 | | 0.99 | | 1.31 | | 1.01 | | 0.89 | | 1.15 | | 0.629 | |  |
|  | | **Q2 vs Q1** | | | | | | **Q3 vs Q1** | | | | | | **Q4 vs Q1** | | | | | | **Q5 vs Q1** | | | | | |  | |
| **Model 3** | | **HR** | | **Lower** | | **Upper** | | **HR** | | **Lower** | | **Upper** | | **HR** | | **Lower** | | **Upper** | | **HR** | | **Lower** | | **Upper** | | **p-trend** | |
|  | |  | |  | |  | |  | |  | |  | |  | |  | |  | |  | |  | |  | |  | |
|  | |  | |  | |  | |  | |  | |  | |  | |  | |  | |  | |  | |  | |  | |
| **n-3 PUFA** | | 1.00 | | 0.87 | | 1.15 | | 0.96 | | 0.84 | | 1.11 | | 0.96 | | 0.81 | | 1.13 | | 0.90 | | 0.79 | | 1.03 | | 0.210 | |
| α-Linolenic acid, ALA (18:3n3) | | 0.96 | | 0.82 | | 1.13 | | 0.87 | | 0.75 | | 1.02 | | 0.85 | | 0.73 | | 0.99 | | 0.75 | | 0.64 | | 0.89 | | <0.001 | |
| Eicosapentaenoic acid, EPA (20:5n3) | | 1.08 | | 0.92 | | 1.27 | | 1.14 | | 0.95 | | 1.36 | | 1.15 | | 0.95 | | 1.40 | | 1.14 | | 0.93 | | 1.41 | | 0.052 | |
| Docosapentaneoic acid, DPA (22:5n3) | | 0.97 | | 0.83 | | 1.14 | | 0.94 | | 0.80 | | 1.11 | | 0.90 | | 0.70 | | 1.15 | | 0.88 | | 0.69 | | 1.14 | | 0.053 | |
| Docosahexaenoic acid , DHA(22:6n3) | | 1.08 | | 0.97 | | 1.19 | | 0.97 | | 0.87 | | 1.09 | | 0.94 | | 0.80 | | 1.09 | | 0.92 | | 0.81 | | 1.05 | | 0.045 | |
|  | |  | |  | |  | |  | |  | |  | |  | |  | |  | |  | |  | |  | |  | |
| **n-6 PUFA** | | 0.94 | | 0.85 | | 1.03 | | 0.85 | | 0.76 | | 0.94 | | 0.84 | | 0.75 | | 0.94 | | 0.74 | | 0.64 | | 0.85 | | <0.001 | |
| Linoleic acid, LA (18:2n6) | | 0.83 | | 0.75 | | 0.91 | | 0.72 | | 0.64 | | 0.80 | | 0.64 | | 0.57 | | 0.73 | | 0.54 | | 0.47 | | 0.61 | | <0.001 | |
| γ-Linolenic acid, GLA (18:3n6) | | 1.19 | | 1.06 | | 1.33 | | 1.49 | | 1.31 | | 1.70 | | 1.69 | | 1.39 | | 2.04 | | 2.04 | | 1.73 | | 2.39 | | <0.001 | |
| Eicosadienoic acid, EDA (20:2n6) | | 0.98 | | 0.89 | | 1.08 | | 0.85 | | 0.74 | | 0.97 | | 0.89 | | 0.80 | | 0.99 | | 0.74 | | 0.61 | | 0.88 | | <0.001 | |
| Dihomo-γ-linolenic acid, DGLA (20:3n6) | | 1.40 | | 1.15 | | 1.72 | | 1.79 | | 1.48 | | 2.15 | | 2.34 | | 1.91 | | 2.88 | | 3.26 | | 2.44 | | 4.36 | | <0.001 | |
| Arachidonic acid, AA (20:4n6) | | 0.93 | | 0.83 | | 1.05 | | 1.10 | | 0.98 | | 1.22 | | 1.04 | | 0.93 | | 1.16 | | 1.03 | | 0.91 | | 1.17 | | 0.083 | |
| Docosatetraenoic acid, DTA (22:4n6) | | 1.06 | | 0.92 | | 1.22 | | 1.16 | | 1.04 | | 1.29 | | 1.27 | | 1.04 | | 1.55 | | 1.49 | | 1.24 | | 1.79 | | <0.001 | |
| Docosapentenoic acid, n6-DPA(22:5n6) | | 1.10 | | 0.87 | | 1.40 | | 1.26 | | 1.01 | | 1.56 | | 1.36 | | 1.05 | | 1.75 | | 1.63 | | 1.06 | | 2.51 | | 0.009 | |
| **Ratios** | |  | |  | |  | |  | |  | |  | |  | |  | |  | |  | |  | |  | |  | |
| 18:3n6 / 18:2n6 (Δ6 desaturase) | | 1.22 | | 1.09 | | 1.37 | | 1.54 | | 1.38 | | 1.71 | | 1.88 | | 1.53 | | 2.31 | | 2.21 | | 1.94 | | 2.50 | | <0.001 | |
| 20:4n6 / 20:3n6 (Δ5 desaturase) | | 0.75 | | 0.67 | | 0.84 | | 0.67 | | 0.61 | | 0.74 | | 0.56 | | 0.49 | | 0.65 | | 0.45 | | 0.36 | | 0.56 | | <0.001 | |
| 20:3n6 / 18:2n6 (DGLA to LA ratio) | | 1.36 | | 1.21 | | 1.52 | | 1.73 | | 1.54 | | 1.94 | | 2.48 | | 2.09 | | 2.94 | | 3.46 | | 2.74 | | 4.38 | | <0.001 | |
| n6 / n3 | | 0.98 | | 0.89 | | 1.08 | | 1.00 | | 0.90 | | 1.11 | | 1.10 | | 0.97 | | 1.25 | | 0.96 | | 0.79 | | 1.17 | | 0.898 | |

Hazard Ratios (HRs) compare quintiles (Q) Q2-Q5 with Q1, where Q1-Q5 were defined by values which divide the distribution of the fatty acid in the overall sub-cohort into fifths.

**Model 1:** Age as underlying time variable, and adjusted for centre, sex, physical activity (inactive, moderately inactive, moderately active, active), smoking (never, former, current), education level (none, primary school completed, technical or professional school, secondary school, longer education), BMI (continuous, kg/m^2^).

**Model 3:** Adjusted as in model 1 + total energy intake (continuous, kcal/day), alcohol (none, ≤6, 6-≤12,12-≤24,>24 g/day), and (continuous, g/d intake of) meat, fruit and vegetables, soft drinks, dairy, fish and shellfish, nuts and seeds, vegetable oil, olive oil, margarine.
