# Supplementary material for: Association of Plasma Phospholipid n-3 and n-6 Polyunsaturated Fatty Acids with Type 2 Diabetes: The EPIC-InterAct Case-Cohort Study
Source: PLoS Med. 2016 Jul 19;13(7):e1002094. doi: 10.1371/journal.pmed.1002094 (PMC4951144; doi:10.1371/journal.pmed.1002094)
Supplement: S5 Table — (DOC) [file pmed.1002094.s010.doc]

| **S5 Table. Study characteristics of prospective studies examining the association of n-3 and n-6 PUFAs with incident T2D** | | | | | | |
| --- | --- | --- | --- | --- | --- | --- |
| **Study*, location, publication** | **Study design; follow-up** | **Demographics:**  **Mean age,**  **%women, %white** | **Total N**  **(n cases)** | **Ascertainment of cases** | **Lipid fraction** | **Adjustments** |
| ARIC, United States†  Wang et al., *Am J Clin Nutr*, 2003;78(1):91-98 | Cohort, 8.1 y | 53.5 y, 46.9%, 100% | 2909 (252) | Serum glucose concentration crossed the diabetes cutoffs (126 mg/dL fasting or 200 mg/dL nonfasting) on a regression line of glucose concentrations by follow-up dates. | Plasma phospholipids, plasma cholesteryl esters† | Age, sex for matching; Baseline BMI, WHR, alcohol intake, cigarette-years of smoking, physical activity, education, and parental history of diabetes |
| MCCS, Australia, Hodge et al., *Am J Clin Nutr*, 2007;86(1):189-197 | Case-cohort, 4 y | 54.5 y, 55.9%, 100% | 3737 (364) | Self-reported, 84% verified by nominated doctors. | Plasma phospholipids | Age, sex, country of birth, family history of diabetes, physical activity, alcohol intake, BMI, and waist-hip ratio |
| VIP, Sweden, Krachler et al., Nutr Metab Cardiovasc Dis, 2008;18(7):503-510 | Nested case-control, 8.8 y | 51.5 y, 56.0%, 100% | 450 (159) | Medical record review according to 1998 WHO criteria | Erythrocyte membrane | Age, sex, survey year, alcohol, BMI, HbA1c |
| CHS, United States, Djousse et al., *Am J Clin Nutr*, 2011;94(2):527-533 | Cohort, 9.6 y | 75.0501 y, 61.1%, 89.2% | 3088 (204) | 1) the new use of insulin or oral hypoglycemic agents, 2)a fasting glucose concentration ≥7 mmol/L (126 mg/dL), or 3)a nonfasting glucose concentration ≥11.1 mmol/L (200 mg/dL). | Plasma phospholipids | Age, race, sex, clinic site, BMI, alcohol consumption, physical activity, and current smoking |
| KIHD, Finland, Virtanen et al., *Diabetes Care*, 2014;37(1):189-196 | Cohort, 19.3 y | 53 y, 0%, 100% | 2212 (422) | 1) Self-reported physician-set diagnosis of type 2 diabetes, 2) fasting plasma glucose ≥7.0 mmol/L 3) 2-h oral glucose tolerance test plasma glucose ≥11.1 mmol/L in follow-up and by national registries | Total serum | Age, examination year, BMI, family history of type 2 diabetes, smoking, education years, leisure-time physical activity, intake of alcohol, serum linoleic acid |
| METSIM, Finland†, Lankinen et al., *Diabetologia*, 2015;58(11):2533-2544 | Cohort, 5.9 y | 54.9 y, 0, 100% | 1302 (71) | Physician’s diagnosis confirmed with a record of anti-diabetic medication use, oral-glucose tolerance test | Total serum, erythrocyte membrane, plasma phospholipids, plasma cholesteryl esters, plasma triglycerides† | Age, BMI, smoking, and physical activity. |
| DPS, Finland, Takkunen et al., Eur J Nutr, 2015 (Epub ahead of print) | Cohort, 11 y | 55.4 y, 67.4%, 100% | 383 (155) | An oral-glucose tolerance test (WHO 1985 criteria, plasma fasting glucose ≥7.8 or 2-hour glucose ≥11.1) and confirmation by a physician | Total serum | Age, sex, study group, smoking, alcohol intake, waist circumference, physical activity at leisure time, study centres |
| EPIC Norfolk,  United Kingdom†, Patel et al., *Am J Clin Nutr*, 2010;95(5):1214-1422 | Nested case-control, 10y | 64 y, 51.6%, 100% | 383 (199) | 1) Self-report of a physician’s diagnosis of diabetes or diabetes medication, 2) General practice diabetes registers, hospital outpatient diabetes registers, and hospital admissions information for diabetes, 3) diabetes-related deaths in the National Death Registry. Self-report without another data source was not qualified as a case. | Plasma phospholipids, erythrocyte membrane | Age, sex, family history of diabetes, BMI, smoking status, physical activity, and alcohol intake |
| EPIC Potsdam,  Germany†, Kroger et al., *Am J Clin Nutr*, 2011;93(3):127-142 | Case-cohort,  7 y | 50 y, 61.3%, 100% | 2724 (673) | Self-repot, diabetes-relevant medication, dietary treatment because of diabetes, verified by questionnaires mailed to the diagnosing physician that asked about the date and type of diagnosis, diagnostic tests, and treatment of diabetes. | Erythrocyte membrane | Age, sex, BMI, waist circumference, cycling, sports activity, education, smoking status, alcohol intake, occupational activity, coffee intake, and fibre intake |

* Abbreviations: ARIC, Atherosclerosis Risk in Communities Study; MCCS, Melbourne Collaborative Cohort Study; VIP, Vasterbotten Intervention Programme; CHS, Cardiovascular Health Study; KIHD, Kuopio Ischaemic Heart Disease Risk Factor Study; EPIC, Epidemiological Investigations into Cancer and Nutrition Study; METSIM, Metabolic syndrome in men; DPS, Diabetes Prevention Study.

† ARIC examined fatty acid profiles of two lipid fractions. METSIM study examined four lipid fractions. In meta-analysis, estimates of phospholipid fractions were used for consistency with the other cohorts. EPIC Norfolk and EPIC Potsdam partly contributed to EPIC InterAct.
