# Supplementary material for: Association of Plasma Phospholipid n-3 and n-6 Polyunsaturated Fatty Acids with Type 2 Diabetes: The EPIC-InterAct Case-Cohort Study
Source: PLoS Med. 2016 Jul 19;13(7):e1002094. doi: 10.1371/journal.pmed.1002094 (PMC4951144; doi:10.1371/journal.pmed.1002094)
Supplement: S6 Table — (DOC) [file pmed.1002094.s011.doc]

| **S6 Table. Meta-analysis results by lipid fraction** **for the prospective associations of circulating fatty acids with incidence of T2D** | | | | | | |
| --- | --- | --- | --- | --- | --- | --- |
| Fatty acids | Fraction* | N studies | HR (95% CI) by fraction† | HR (95% CI) overall‡ | I^2^ crude | I^2^ adjusted for fraction§ |
| **n3 PUFA** |  |  |  |  |  |  |
| Total n3 | Total serum | 2 | 0.83 (0.73, 0.95) |  |  |  |
|  | Erythrocytes | 2 | 0.99 (0.90, 1.09) |  |  |  |
|  | Phospholipids | 1 | 1.10 (0.95, 1.27) | 0.95 (0.84, 1.06) | 63.2 | 0.0 |
| 18:3n3 | Total serum | 2 | 1.01 (0.88, 1.17) |  |  |  |
|  | Triglycerides | 1 | 0.83 (0.63, 1.09) |  |  |  |
|  | Chol. esters | 2 | 0.89 (0.78, 1.02) |  |  |  |
|  | Erythrocytes | 3 | 0.97 (0.88, 1.06) |  |  |  |
|  | Phospholipids | 4 | 0.93 (0.88, 0.99) | 0.96 (0.92, 0.99) | 0.0 |  |
| 20:5n3 | Total serum | 2 | 0.86 (0.66, 1.11) |  |  |  |
|  | Triglycerides | 1 | 1.01 (0.80, 1.28) |  |  |  |
|  | Chol. esters | 2 | 1.00 (0.92, 1.08) |  |  |  |
|  | Erythrocytes | 4 | 0.92 (0.83, 1.01) |  |  |  |
|  | Phospholipids | 4 | 1.02 (0.95, 1.09) | 0.96 (0.90, 1.01) | 38.7 | 31.6 |
| 22:5n3 | Total serum | 2 | 0.84 (0.68, 1.04) |  |  |  |
|  | Triglycerides | 1 | 0.74 (0.55, 0.99) |  |  |  |
|  | Erythrocytes | 3 | 0.95 (0.88, 1.04) |  |  |  |
|  | Phospholipids | 2 | 0.96 (0.78, 1.19) | 0.92 (0.86, 0.99) | 38.2 | 44.9 |
| 22:6n3 | Total serum | 3 | 0.90 (0.83, 0.97) |  |  |  |
|  | Triglycerides | 1 | 0.99 (0.77, 1.27) |  |  |  |
|  | Chol. esters | 2 | 0.99 (0.91, 1.09) |  |  |  |
|  | Erythrocytes | 4 | 1.02 (0.93, 1.12) |  |  |  |
|  | Phospholipids | 4 | 1.00 (0.92, 1.10) | 0.97 (0.91, 1.03) | 52.6 | 56.4 |
|  |  |  |  |  |  |  |
| **n-6 PUFA** |  |  |  |  |  |  |
| Total n6 | Total serum | 2 | 0.84 (0.58, 1.22) |  |  |  |
|  | Erythrocytes | 2 | 0.93 (0.85, 1.03) |  |  |  |
|  | Phospholipids | 1 | 0.93 (0.80, 1.09) | 0.89 (0.77, 1.02) | 73.9 | 82.4 |
| 18:2n6 | Total serum | 3 | 0.96 (0.84, 1.10) |  |  |  |
|  | Triglycerides | 1 | 0.82 (0.63, 1.06) |  |  |  |
|  | Chol. esters | 2 | 0.82 (0.71, 0.95) |  |  |  |
|  | Erythrocytes | 4 | 0.82 (0.68, 0.97) |  |  |  |
|  | Phospholipids | 3 | 0.79 (0.70, 0.89) | 0.86 (0.77, 0.95) | 50.1 | 25.9 |
| 18:3n6 | Triglycerides | 1 | 1.09 (0.86, 1.38) |  |  |  |
|  | Chol. esters | 2 | 1.05 (0.89, 1.23) |  |  |  |
|  | Erythrocytes | 2 | 1.17 (0.95, 1.45) |  |  |  |
|  | Phospholipids | 1 | 1.12 (0.97, 1.30) | 1.19 (1.08, 1.30) | 0.0 |  |
| 20:2n6 | Erythrocytes | 2 | 0.82 (0.54, 1.23) |  |  |  |
| 20:3n6 | Total serum | 1 | 1.03 (0.87, 1.22) |  |  |  |
|  | Triglycerides | 1 | 1.12 (0.90, 1.40) |  |  |  |
|  | Chol. esters | 2 | 1.14 (0.97, 1.34) |  |  |  |
|  | Erythrocytes | 3 | 1.19 (1.06, 1.33) |  |  |  |
|  | Phospholipids | 3 | 1.49 (1.21, 1.83) | 1.30 (1.11, 1.52) | 77.0 | 59.7 |
| 20:4n6 | Total serum | 1 | 0.79 (0.67, 0.93) |  |  |  |
|  | Triglycerides | 1 | 0.88 (0.69, 1.12) |  |  |  |
|  | Chol. esters | 2 | 1.12 (0.98, 1.29) |  |  |  |
|  | Erythrocytes | 4 | 0.99 (0.87, 1.14) |  |  |  |
|  | Phospholipids | 3 | 1.15 (1.05, 1.27) | 1.02 (0.91, 1.16) | 71.4 | 0.0 |
| 22:4n6 | Erythrocytes | 3 | 1.23 (0.93, 1.64) |  |  |  |
|  | Phospholipids | 1 | 1.14 (1.07, 1.21) | 1.15 (0.94, 1.41) | 60.2 |  |
| 22:5n6 | Phospholipids | 1 | 1.22 (0.97, 1.53) | 1.22 (0.97, 1.53) |  |  |
| **Ratios** |  |  |  |  |  |  |
| 18:3n6/18:2n6 | Total serum | 1 | 0.94 (0.79, 1.11) |  |  |  |
|  | Triglycerides | 1 | 1.14 (0.91, 1.43) |  |  |  |
|  | Chol. esters | 1 | 1.26 (0.18, 8.91) |  |  |  |
|  | Phospholipids | 1 | 1.60 (1.29, 1.99) | 1.22 (0.73, 2.06) | 93.0 | 84.2 |
| 20:4n6/20:3n6 | Total serum | 1 | 0.79 (0.66, 0.95) |  |  |  |
|  | Triglycerides | 1 | 0.74 (0.57, 0.97) |  |  |  |
|  | Chol. esters | 1 | 0.88 (0.47, 1.65) |  |  |  |
|  | Erythrocytes | 3 | 0.80 (0.67, 0.97) |  |  |  |
|  | Phospholipids | 3 | 0.75 (0.66, 0.85) | 0.77 (0.70, 0.84) | 0.0 |  |
| 20:3n6/18:2n6 | Erythrocytes | 3 | 1.41 (1.17, 1.70) |  |  |  |
|  | Phospholipids | 1 | 1.91 (1.55, 2.34) | 1.56 (1.30, 1.89) | 40.0 | 0.0 |
| n6/n3 | Phospholipids | 1 | 0.93 (0.80, 1.07) | 0.93 (0.80, 1.07) |  |  |

* Studies assessed relative concentrations of each fatty acid (% total fatty acids) of different lipid fractions. Chol. stands for cholesteryl. Triglycerides, cholesteryl esters, and phospholipids, if used, were taken from plasma samples in each study. Erythrocytes means erythrocyte membrane.

† Hazard ratio (HR) and 95% confidence interval (CI) were estimated per study-specific standard deviation for each lipid fraction. If a single study assessed fatty acids in different lipid fractions, multiple estimates by fraction were included in the fraction-specific HR. For the overall HR, only one estimate from each study was included.

‡ HR and 95% CI were estimated per study-specific standard deviation. See the main table (Table 3) for the number of studies evaluated in the analysis for each fatty acid (one estimate from each study).

§ I^2^ was calculated after controlling for a categorical variable of lipid fractions. None of the categories for any fatty acids showed statistically significant heterogeneity owing to lipid fraction. Adjusted I^2^ were not calculated when crude I^2^ were zero or when multiple fractions were not used across studies or when meta-regression needed to pool multiple fractions from a single cohort.
