# Supplementary material for: Association of Plasma Phospholipid n-3 and n-6 Polyunsaturated Fatty Acids with Type 2 Diabetes: The EPIC-InterAct Case-Cohort Study
Source: PLoS Med. 2016 Jul 19;13(7):e1002094. doi: 10.1371/journal.pmed.1002094 (PMC4951144; doi:10.1371/journal.pmed.1002094)
Supplement: S7 Table — (DOC) [file pmed.1002094.s012.doc]

**S7 Table. Associations of plasma phospholipid n-3 and n-6 PUFAs with incidence of T2D for Models 1, 2 and 3**

| **HR (95% CI)** | **Model 1** | **Model 2** | **Model 3** |
| --- | --- | --- | --- |
|  |
|  |  |  |  |
| **n-3 polyunsaturated fatty acids** | 0.97 (0.94, 1.01) | 0.98 (0.93, 1.03) | 0.98 (0.93, 1.03) |
| α-Linolenic acid, ALA (18:3n3) | 0.92 (0.88, 0.97) | 0.93 (0.88, 0.98) | 0.93 (0.88, 0.98) |
| Eicosapentaenoic acid, EPA (20:5n3) | 1.02 (0.99, 1.06) | 1.04 (1.01, 1.07) | 1.05 (0.99, 1.10) |
| Docosapentaneoic acid, DPA (22:5n3) | 0.94 (0.91, 0.98) | 0.95 (0.91, 0.99) | 0.95 (0.91, 1.00) |
| Docosahexaenoic acid, DHA (22:6n3) | 0.95 (0.91, 1.00) | 0.95 (0.90, 1.01) | 0.95 (0.90, 1.01) |
|  |  |  |  |
| **n-6 polyunsaturated fatty acids** | 0.91 (0.87, 0.94) | 0.89 (0.85, 0.93) | 0.87 (0.83, 0.91) |
| Linoleic acid , LA(18:2n6) | 0.83 (0.80, 0.86) | 0.81 (0.78, 0.84) | 0.80 (0.77, 0.83) |
| γ-Linolenic acid, GLA (18:3n6) | 1.19 (1.13, 1.25) | 1.19 (1.14, 1.24) | 1.19 (1.14, 1.24) |
| Eicosadienoic acid, EDA (20:2n6) | 0.91 (0.88, 0.95) | 0.90 (0.86, 0.94) | 0.89 (0.85, 0.94) |
| Dihomo-γ-linolenic acid, DGLA(20:3n6) | 1.44 (1.32, 1.57) | 1.45 (1.32, 1.59) | 1.46 (1.34, 1.59) |
| Arachidonic acid, AA (20:4n6) | 1.02 (0.99, 1.06) | 1.02 (0.98, 1.07) | 1.02 (0.98, 1.06) |
| Docosatetraenoic acid, DTA (22:4n6) | 1.13 (1.07, 1.20) | 1.13 (1.06, 1.20) | 1.13 (1.06, 1.21) |
| Docosapentenoic acid, n6-DPA (22:5n6) | 1.13 (1.05, 1.22) | 1.13 (1.05, 1.23) | 1.14 (1.05, 1.24) |
|  |  |  |  |

Hazard Ratios (HRs) for EPIC-InterAct are per 1 standard deviation of each fatty acid for models 1, 2 and 3.

Model 1: included age (as underlying timescale) and adjusted for centre, sex, physical activity index (inactive, moderately inactive, moderately active, active), smoking status (never, former, current), education level (none, primary school, technical or professional school, longer duration education) and BMI (continuous).

Model 2: Adjusted as in model 1 + total energy intake (continuous), alcohol (none, ≤6, 6-≤12,12-≤24,>24 g/day) and (continuous, g/d intake of) meat, fruits, vegetables, dairy products, and soft drinks.

Model 3: Adjusted as in model 2 + (continuous, g/d intake of) fish and shellfish, nuts and seeds, vegetable oil, olive oil, and margarine.
