# Supplementary material for: Association of Plasma Phospholipid n-3 and n-6 Polyunsaturated Fatty Acids with Type 2 Diabetes: The EPIC-InterAct Case-Cohort Study
Source: PLoS Med. 2016 Jul 19;13(7):e1002094. doi: 10.1371/journal.pmed.1002094 (PMC4951144; doi:10.1371/journal.pmed.1002094)
Supplement: S1 Text — (DOC) [file pmed.1002094.s013.doc]

**S1 Methods.**

*Systematic review and meta analysis of published literature*

The review protocol is available upon request. To compare the findings from EPIC-InterAct with the published literature we identified prospective studies that reported on the association of circulating n-3 or n-6 PUFAs with T2D using PubMed, and by scanning the reference lists of articles identified for relevant studies and reviews. The search terms were related to n-3 and n-6 PUFAs (“polyunsaturated fatty acids” OR “n-3” OR “n-6” OR “α-linolenic” OR “eicosapentaenoic” OR “docosapentaenoic” OR “docosahexaenoic” OR “linoleic” OR “γ-linolenic” OR “eicosadienoic” OR “dihomo-γ-linolenic” OR “arachidonic” OR “docosatetraenoic” OR “docosapentenoic”) and diabetes risk (“diabetes”) in prospective study design (“prospective” OR “cohort” OR “nested”). Prospective studies published by 3rd November 2015 were included with no limits on publication date or language. We included studies that measured circulating (serum, plasma, erythrocyte, or whole blood) n-3 or n-6 PUFAs at baseline, ascertained incident T2D, and provided a relative risk and corresponding 95% CI for T2D in relation to circulating n-3 or n-6 PUFA levels. We excluded studies without prospective design or examining only dietary PUFAs. For each contributing study, information was extracted according to a pre-specified protocol by two authors independently. The results were reported as odds ratio, risk ratios, or hazard ratios and were assumed to be approximate estimates of the relative risk.
