# Supplementary material for: Association of Plasma Phospholipid n-3 and n-6 Polyunsaturated Fatty Acids with Type 2 Diabetes: The EPIC-InterAct Case-Cohort Study
Source: PLoS Med. 2016 Jul 19;13(7):e1002094. doi: 10.1371/journal.pmed.1002094 (PMC4951144; doi:10.1371/journal.pmed.1002094)
Supplement: S2 Text — (PDF) [file pmed.1002094.s014.pdf]

EPIC-InterAct: associations between plasma phospholipid non-saturated fatty acids and incident T2D

Analysis Plan, August 2014

Introduction
This is the plan for the analysis of the association of measured plasma phospholipid PUFAs with incident T2D using EPIC-InterAct data. All analyses will be performed by Stephen Sharp.

Exclusions
Individual fatty acids (FAs) with a mean concentration < 0.05% in the subcohort will not be analysed.

Additional FA exposures
As well as each individual FA, the following groupings will be analysed:

- Total n-3 PUFA.
- Total n-6 PUFA.
- Total trans fatty acids.
- Sum: 20:5n3 + 22:5n3 + 22:6n3 (this is to separate marine-origin vs. non-marine).
- Ratio: 20:5n3 / 20:4n6.
- Ratio: 18:3n6 / 18:2n6.
- Ratio: 18:3n3 / 18:2n6.
- Ratio: 20:4n6 / 20:3n6.
- Ratio: (total n-6 PUFA) / (total n-3 PUFA).

Descriptive information
The distribution (mean, SD) of each FA exposure will be summarised by:

- country.
- age at baseline (<40, 40-<60, ≥60 years).
- sex.
- BMI (<25, 25-<30, ≥30 kg/m^2).

Associations between FA and incident T2D
Hazard ratios and 95% CIs will be estimated for each FA exposure using the following approaches:

- per 1 SD increase.
- quintile 2 vs quintile 1.
- quintile 3 vs quintile 1.
- quintile 4 vs quintile 1.
- quintile 5 vs quintile 1.

SDs and cut-offs for quintiles will be calculated using the data in the subcohort. Conjugated linoleic acid (c18:2n6t) does not have sufficient variability in some countries to allow categorization into quintiles, so will be been analysed in tertiles instead.
Hazard ratios and 95% CIs will be estimated using Prentice-weighted Cox regression models, fit separately within each country and combined across countries using random effects meta-analysis. The following main models will be fit:

Model 1 – adjusted for centre, age (as the underlying timescale), sex, physical activity, smoking, education level, BMI.

Model 2 – model 1 + total energy intake, alcohol (5 categories – 0 g/day, >0-6 g/day, >6-12 g/day, >12-24 g/day, ≥24 g/day), meat, fruit and vegetables, soft drinks, dairy, fish and shellfish, nuts and seeds, vegetable oil, olive oil, margarine

A p-value for linear trend across quintiles (or tertiles where applicable) will be calculated for each model.

The impact of HRT and oral contraceptive pill use on the results will be investigated by menopausal status too (4 categories).

The following **sensitivity analyses** will be performed for the per 1 SD analyses only:

To test the effect of co-adjustment for other fatty acids:

Model 2a - model 2 + c14:0, c16:0, c18:0.
Model 2b - model 2 + c15:0, c17:0.
Model 2c - model 2 + c20:0, c22:0, c23:0, c24:0.

To assess any potential impact of prevalent disease at baseline:
Model 2d – model 2 + prevalent MI, stroke and cancer (all yes/no variables).

To assess potential reverse causality:
Model 2e – model 2 + baseline HbA1c.
Model 2f - model 2 excluding individuals with baseline HbA1c≥6.5%.
Model 2g – model 2 excluding individuals with incident T2D diagnosed within the first 2 years after baseline.

Amendment following working group review of first draft of manuscript, December 2015

Re-label Model 2 to be Model 3.
Add a new Model 2 which is the same as the original Model 2 but without fish and shellfish, nuts and seeds, vegetable oil, olive oil, margarine.
